# Cultural adaptation of self-management of type 2 diabetes in Saudi Arabia (qualitative study)

**DOI:** 10.1371/journal.pone.0232904

**Published:** 2020-07-28

**Authors:** Thamer Al Slamah, Barbara I. Nicholl, Fatima Y. Alslail, Leanne Harris, Craig A. Melville, Deborah Kinnear

**Affiliations:** 1 Human Health Department, College of Applied Medical Sciences, Qassim University, Kingdom of Saudi Arabia, Riyadh, Saudi Arabia; 2 General Practice and Primary Care, Institute of Health and Wellbeing College of Medicine, Veterinary and Life Science, University of Glasgow, Glasgow, United Kingdom; 3 Director of the National Diabetes Control and Prevention Program, Ministry of Health, Kingdom of Saudi Arabia, Riyadh, Saudi Arabia; 4 School of Medicine, Dentistry & Nursing, University of Glasgow, Glasgow, United Kingdom; 5 Mental Health and Wellbeing, Institute of Health and Wellbeing College of Medicine, Veterinary and Life Science, University of Glasgow, Glasgow, United Kingdom; Chinese Academy of Medical Sciences and Peking Union Medical College, CHINA

## Abstract

**Background:**

Saudi Arabia is continuously working on developing its health care system, however with the high prevalence of type 2 diabetes and comorbidities, such as cardiovascular diseases, self-management education programmes are essential. As part of a planned series of studies to develop a culturally sensitive type 2 diabetes self-management programme, this study explores the need versus barriers and facilitators relevant to implementing a national programme for type 2 diabetes self-management education within the community and health care system in Saudi Arabia.

**Methods:**

A qualitative methodology was used to explore the views of a multidisciplinary group of diabetes health professionals and adult patients with type 2 diabetes. The views of nine health professionals working at a specialised diabetes care centre were gathered at two focus groups (four and five) that included doctors, nutritionists, health educators and nurses. Individual interviews with 12 patients with type 2 diabetes (six females and six males) attending the centre were also carried out. Recurring themes through the translated transcripts were studied and treated by the research group under pre-set protocols.

**Results:**

Focus groups with health professionals revealed three main themes. 1. Resources: availability of resources and how they impacted on performance and patients’ care; 2.Familiarity with self-management education programmes: educating patients and raising awareness among them; and 3. Lifestyle: patients’ lifestyle and how it could affect their compliance with self-management programmes. Interviews with patients also revealed three main themes. 1. Habits: post diagnosis changes in patients’ attitudes and behaviours towards diet and physical activity; 2. Health education: awareness of managing type 2 diabetes through health centre advice or self-education; and 3. Culture and society: a lack of cultural or social support created by some social practices or conventions.

**Conclusion:**

The findings from this study highlight a gap in type 2 diabetes care system that can be breached through the development of a Saudi specific self-management programme for type 2 diabetes. The identified barriers and facilitators can be used for adapting a self-management programme to the Saudi context. However, initial training is needed for local health professionals to understand the mechanisms of self-management programmes. Such programmes will need to infiltrate to the society, and the patients’ families, in particular to tackle the rising prevalence of type 2 diabetes in Saudi Arabia and provide a friendlier, more supportive environment for the current patients to self-manage their diabetes.

## Background

The risk of developing type 2 diabetes or having a poor prognosis of the condition correlates with a number of behavioural factors such as obesity, sedentary lifestyle, smoking and unhealthy diet [1]. Diabetes self-management programmes have been shown to improve glycaemic control and decrease diabetic complications [2, 3]. There are no well-developed self-management programmes in Saudi Arabia. However, our recent systematic review suggested that type 2 diabetes self-management programmes may be effective in improving glycaemic control in patients with type 2 diabetes living in the Gulf countries [4]. Type 2 diabetes self-management programmes support individuals to adopt healthy lifestyle changes into their daily routine such as increasing physical activity and improving dietary habits. However, a key attribute for the success of self-management programmes is their suitability for the cultural and social environment that they are running in [2, 3, 5]. To date, none of the studies investigating type 2 diabetes self-management programmes for implementation has taken into account the cultural context in Saudi Arabia, although some of these programmes were independently set-up in Saudi Arabia [4].

In the UK, diabetes education and self-management for ongoing and newly diagnosed patients is standardised through a national programme (DESMOND). Similarly, in the USA diabetes self-management education (DSME) is a progressive strategy that enables individuals with pre-diabetes or diabetes to gain control over the progress of diabetic complications and positively contribute to their health care [6]. DSME was initially introduced in the USA through ethnicity-specific programmes, and was later adopted by other English speaking countries [7]. Some other countries or societies launched the programme after adapting it to the local social environment, including appropriate translation, as in the USA with pacific island and Latino communities [7, 8]. DSME can improve glycaemic control as evidenced by a reported 1% reduction of glycated haemoglobin (HbA1c), which reflects higher patients’ compliance, and healthy adjustments over eight weeks or more [9]. Stern control of blood glucose level can contribute in delaying or mitigating diabetic complications such as nephropathy, peripheral neuropathy and visual impairment, reducing the risk of heart disease or stroke among diabetic patients to near general population average levels [2, 3]. The changes in lifestyle provided by DSME may also benefit individuals known to be at elevated risk of developing diabetes, and it is wise to include them in such a programme [4]. Similar benefit of DSME was observed from some self-management education initiatives in Saudi Arabia and other gulf countries. However, the success of these initiatives was limited by lack of two key elements in well-developed systematic programmes, which are cultural adaptation and model repeatability [4].

The health system in Saudi Arabia is based on national health insurance, which is provided to all Saudi citizens, and some of the residents. This free of charge service allows the introduction of patient education programmes such as diabetes self-management at a national standard [10]. Large cities, such as Riyadh, Jeddah, Buriaydah and many others host highly equipped and specialised hospitals, which provide the tertiary level of health care (advanced specialities, major surgeries and consultancy). The primary care units, such as general practices, are extensively distributed within cities, villages and remote areas. These provide a general practitioner service and are equipped with their own laboratories and radiology department [11]. At the secondary care level, are the specialised centres, which include endocrine centres that provide care for patients with diabetes among others. Health care at these centres is provided by endocrinologists, nutritionists, specialised nurses and health educators [11]. In addition, the Saudi ministry of health (MOH) has introduced both electronic and phone services since 2017/18, through which patients can receive tailored and confidential clinical advice and prescriptions when applicable [11].

The estimated 33 million Saudi population has a growth rate of 3.2% per year [12] reaching near 40 million by 2025 as per the United Nations projections [13]. More than 67% are under the age of 30 years and only 5% are above 60 years. Males and females are approximately equal in number, with a life expectancy of 72.5 years in males compared to 74.7 years in females [12]. The highest incidence of type 2 diabetes in Saudi Arabia is in individuals who are in their sixth decade and is greater among females than males and is higher in individuals with a higher BMI [14]. In the 2010 census, 2.5 million individuals (9.2%) had diabetes [12] with a predicted increase rate of 200,000 per year. According to population projections, more than 13% and up to 20% either will have diabetes or be at risk [15]. Perhaps 89% to 97% of all patients with diabetes visiting a given centre will be of type 2 diabetes [16,17].

Deciding on the standards and the quality of the programmes that diabetes educators are offering for individuals under their care is difficult without previous knowledge of the suitability of such programmes to the local environment and culture [5, 18]. The development of national standards in any country, including Saudi Arabia, requires investigating the views and readiness of practitioners and patients [19]. In general, patients with diabetes may lack self-esteem or can be cautious towards performing physical exercise or dietary control [20]. Some doubt the benefit of doing so or believe that their medical condition would prevent them from doing so [21]. Personal, socioeconomic and cultural factors may impose further limitations. The effect of these factors will vary according to gender, age, family obligations, work responsibilities, income, residence, education, in addition to their general and diabetic-associated health status [21, 22]. The process of implementing international programmes such as DSME into a new local environment may require a relatively extended period of time to allow careful examination of local social and official health policies, economic status and resources and also to thoroughly understand how to normalise the practices of these programmes within the local cultural context, which can only be achieved by high quality social and behavioural research [23, 24].

The current qualitative study aimed to assess factors that could assist in, or hinder the implementation of a diabetes self-management programme in Saudi Arabia, and alterations needed to allow self-management programmes to be culturally acceptable to patients.

## Methods

### Research design

In order to assess the need and benefit of cultural adaptation, the study design followed the earlier stage of Kumpfer’s cultural adaptation model [24]. This model represents a logical framework for conducting such research. Kumpfer suggested nine steps for the cultural adaptation of health programmes starting with identifying the needs, assessing them within a reflective population sample, discussing how to tackle them through relevant focus groups, piloting the focus group recommendations, using the outcome of the pilot study to improve the quality and support of the programme, revising the programme for any additional requirements or alterations before concluding the final evaluation prior to dissemination of results and publication [24]. In order to “identify the needs”, the first step in Kumpfer model, we have previously carried out a systematic review of pilot studies in Saudi Arabia and other gulf countries on type 2 diabetes self-management to assess their methods and if the subjects of any of these studies achieved better control of their condition [4]. The current study employed a qualitative design (focus groups and interviews) to explore the “needs” further through the views of patients with type 2 diabetes and the professionals responsible for their treatment and care on what is “needed” in the type 2 diabetes patient journey, what is specific to Saudi community and how can this fit self-management programmes or can be fitted to them.

### Approach

This qualitative study is meant to study the specific needs of individuals with type 2 diabetes. This approach is called by some qualitative researchers (phenomenological) [25], as it is concerned with a specific phenomenon. This phenomenon is studied within part of the local Saudi community, which further classifies the approach of the study as cultural or ethnographic [25]. In order to candidate the background of participants within the settings of these two approaches we have identified the following pairs: common experience (type 2 diabetes) versus common culture (Saudi community). This pair was represented by Saudi type 2 diabetes patients. The second pair was common involvement (management of type 2 diabetes) versus common environment (Saudi health system). This pair was represented by the health professionals at a Saudi specialised health centre. To ensure association between the two pairs the patients and the health professionals were of the same health centre.

Taking into consideration the small sample size, and the limitation of the study to one centre, this study did not aim to follow a grounded theory approach, instead themes that are common and shared among participants are used in a thematic analysis model [26]. The key difference is that a grounded theory approach would have been more refined due to inclusion of participants of wider geographical and epidemiological base, and would have included theoretical sampling from previous and current social studies [26].

### Validity and saturation

The sample size was measured based on the pool, which was limited to one health centre, and repetitions of concepts at early collection stage (saturation). For the purpose of the study, triangulation was considered sufficient to establish validity based on consensus between the two focus groups, among the 12 patients and the focus groups versus the patients’ interviews [27]. The patients interviews provided further validity as each patient was interviewed separately, but all asked the same questions in the same order.

The authors may have some bias towards self-management approach based on their convention of its success elsewhere outside Saudi Arabia. However, we were keen to actively search in the manuscripts for “hints” that would suggest differently, whether in the focus groups or in the patients’ interviews.

The authors of this work were divided at each stage as follows: ethical approval application (TA, BN, CM), data collection (TA), checking data collection ethics (CM, FA), initial data analysis (TA), secondary data analysis (TA, LH, DK), data analysis verification (DK, BN), final judge (BN, CM).

### Study population

The study population were health professionals at Buraydah Endocrine and Diabetes Centre in King Fahd Specialist Hospital, Qassim, Saudi Arabia to share their experience versus patient experience; and patients with type 2 diabetes attending the centre’s clinics to be asked about their experience. All, who gave their consent were enrolled in the study. All completed their participation to a satisfactory level (remain engaged and answered all questions or discussed thoughts of interest).

### Data collection

#### Health professionals

Two focus groups were carried out to explore implementable aspects, feasibility and value of self-management programmes among their patients as well as challenges. One was formed of four and another of five professionals. Each of the focus groups included at least one doctor, nurse, nutritionist and health educator. The latter had a bachelor’s degree in health education and was certified as a diabetes health educator for their speciality. All health professionals had at least one year of experience in providing care for patients with type 2 diabetes and at least one year of that work experience was in Saudi Arabia.

The head of research at the Diabetes Centre, passed the invitation to the 10 doctors, four health educators, two nutritionist and 20 nurses working at the centre to attend the focus group at one of two available slots, on two separate days, each three hours, during working hours. Those who expressed willingness and availability were allocated to the time slot of their choice. Both groups were carried out in the same lecturing room at the centre.

#### Patients

The aim of the patient interviews was to elicit potential attitudes and behaviours towards common items within self-management programmes and the appropriate educational approach that can facilitate acquiring favourable health behaviours. No particular selection criteria were applied to approaching patients. All patients who were above 18 years old and present in the outpatient clinic were potential candidates. Flyers with study information were handed over to the patients over three weeks, and all those who responded and consented to take part, at no incentive, were recruited after a thorough explanation of the study. Interviews were carried out in simple Arabic language to ensure accessibility for all patients. All patients had type 2 diabetes (diagnosed for one year or more), were over 18 years old, lived locally, attended the outpatient clinic and could speak, read and write Arabic. None of the patients had any communication difficulties or disabilities. Twelve patients took part in the interviews. All patients were interviewed at the same counselling room (a reasonably sized quiet room with a desk and 4 chairs). Each interview lasted for almost one hour.

### Ethical approval and informed consent

Ethical approval (number: H-04-Q-001) was granted by MOH in Saudi Arabia, on 09/01/2018 and the University of Glasgow (number: 200170169), College of Medicine, Veterinary and Life Science Research Ethics Committee, on 09/08/2018.

All participants gave their informed written consent and agreed to be audio-recorded. Information sheets about this study, were provided in the Arabic language to potential participants. The information sheet and consent form were also verbally communicated.

### Moderator guidelines

The moderator’s guides and interview schedule for focus groups and patient interviews (S1 Appendix) were developed based on a review of the literature carried out by the study team, particularly in relation to the DESMOND intervention approach, a training programme aimed at supporting participants to become experts themselves in diabetes self-management training and education [19]. The moderator (TA) is bilingual (Saudi Arabic and English) and has experience of working with patients with type 2 diabetes and health professionals in Saudi Arabia. The moderator relied on a voice recorder to record the conversations, while he took field notes.

In the focus group, the moderator agreed with the participants for himself to start with an opening paragraph, or highlights, rather than a question, for them to share their ideas “whatever come to their mind” around the topic, from one to another. The moderator at few occasions would use body gesture or eye contact to encourage a particular participant to talk.

### Translation

The focus groups and interviews were transcribed in Arabic. Two different experienced translators, of no less than five years as judicial translators, received copies of the original Arabic transcripts and translations were verified by a third. The translators were requested to translate verbatim (word for word) the written transcripts. As the translations were judged to be identical, the rest of the transcripts were then distributed among the two translators (a transcript of one focus group and six transcripts of patient interviews each) [28]. The moderator (TA) also reviewed audio transcriptions to ensure that translations were as accurate as possible. Due to the direct verbatim translation, some quotes required some adjustment, as in the following example:

An original verbatim translation of a quote from a health professional:

“*we began to do a consulting course for diabetologists who are being in the centre now*”

Adjusted quote *“We began a consulting course for diabetologists who are now in the centre”* (FG1-MD)

### Data analysis (methods and interpretations)

The qualitative data software analysis package, Atlas/Ti software 18 (Scientific Software Development GmbH, Berlin, Berlin-Brandenburg, Germany) was used to organise the English transcripts in order to analyse the data. The data analysis was divided into the four stages of a thematic framework method [29]. At the first stage, symbolic domains that gave distinctive meaning within the transcripts were identified. The second stage aimed at identifying domains that shared a common pattern. The third stage involved investigating the crude themes among the domains to start producing codes. Each of these codes reflected a component of one investigated inquiry for any of the themes. The participants’ “hints” / views were used to classify these codes as facilitators versus barriers. Once initial codes were generated, discussions took place within the research team to identify the best approach by which these codes could be classified into families / refined subthemes according to their similarity and their contextual ability to address the main research questions. Finally, Atlas/Ti software 18 was used to manage the analysis of the final codes.

Based on the approach of this qualitative study, the emerging themes from the discussion in the focus groups, or patients’ answers to interviews’ questions described the patient daily journey in managing type 2 diabetes (phenomenon) and how that can be specific to the Saudi community or Saudi health system (culture) [26].

**Fig 1:**
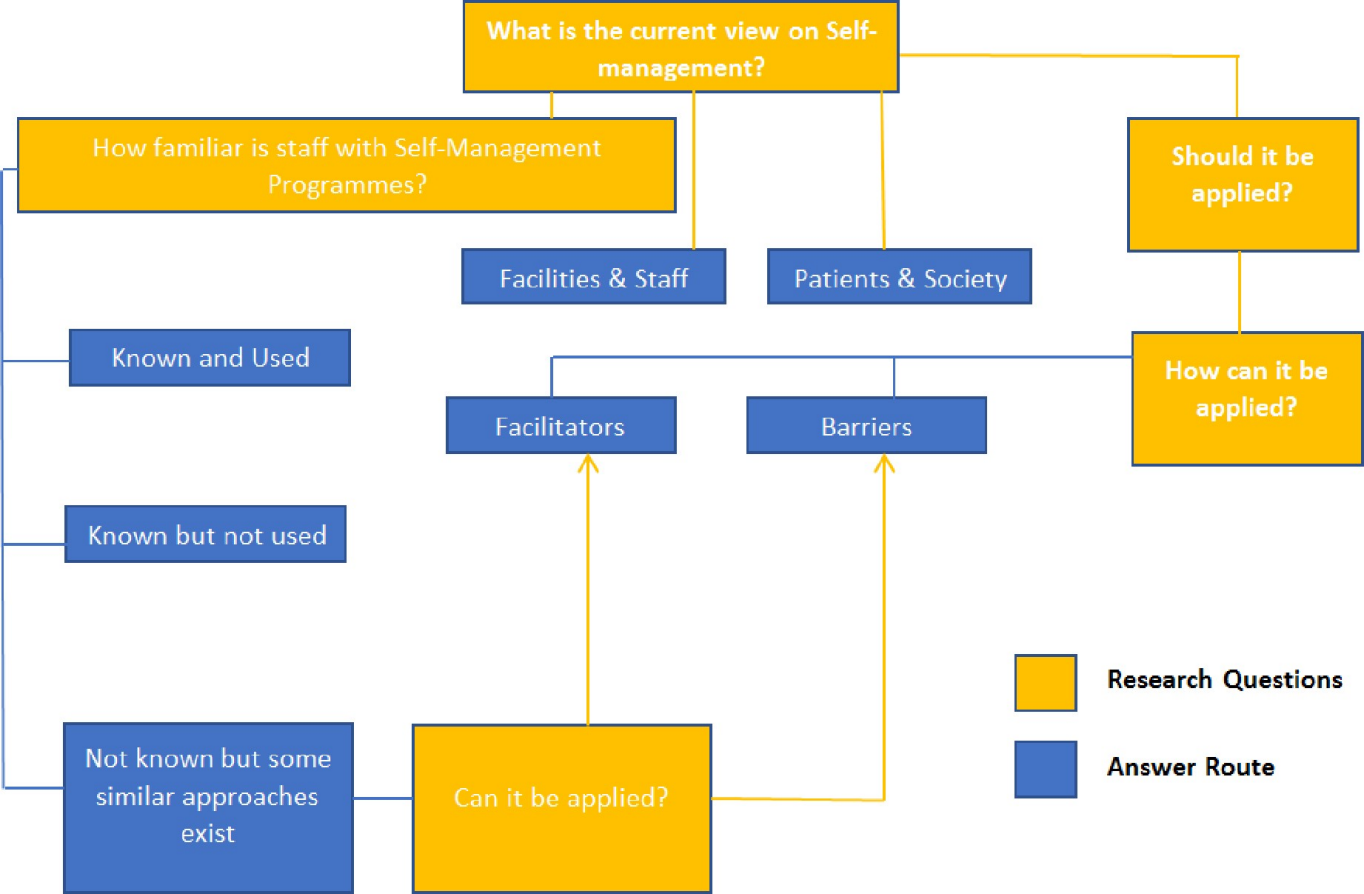
Thematic map development. The themes in the study developed upon seeking answers for the main research questions of the study (in yellow), which aimed to identify the participants’ view (in blue) on self-management programme. First, we assessed the participants feed-back on how familiar they are with these programmes, particularly the health professionals. Then answers for sub-research questions were sought. These questions aimed to assess the need for, feasibility of and approach to be taken to implement a self-management programme. On identifying answers for these questions, further themes and subthemes had emerged. Within the subthemes, factors that can help (facilitators) or restrict (barriers) the development of a self-management programme could be identified.

Fig 1 provides the theme seeking approach. Based on repetitions and shared concepts, the data deemed sufficient and no further recruitment was required.

## Results

Qualitative analysis of the transcripts revealed prominent themes in the focus groups and patient interviews. The themes were represented by frequency and exact quotes, which are provided in *italic*.

### Health professional focus groups

Table 1 presents the participants and identifiers for each individual within each of the two focus groups.

**Table 1:** Keys for participants’ Identification.

**Focus Group Number 1**	
Male Doctor	FG1-MD
Male Nutritionist	FG1-MNT
Female Nurse 1	FG1-FNR1
Female Nurse 2	FG1-FNR2
Female Health Educator	FG1-FHE
**Focus Group Number 2**	
Female Doctor	FG2-FD
Female Nutritionist	FG2-FNT
Female Nurse number	FG2-FNR
Female Health Educator	FG2-FHE
**Patients’ Interviews**	
Example 1: Male Patient 1	MP-1
Example 2: Female Patient 1	FP-1

Three main themes that surrounded the essential elements of establishing or adapting self-management education programmes emerged from the data (Table 2). These were ‘resources’, ‘familiarity with self-management education’ and ‘lifestyle’. Within each theme, subthemes are used to describe the barriers and facilitators for implementation of self-management education programmes in Saudi Arabia that were identified through the data analysis.

**Table 2:** Themes and subthemes from health professional focus group.

	Subthemes	
Theme	Facilitators	Barriers
Resources	Qualified experienced staff teams	High number of patients
		Health centre location
	Educating patients through different communication channels	
		Strategy for patient follow -up
		Financial constraints
Familiarity with self-management education programmes	One to one tailored appointments for patient education	Lack of previous systematic application
	Patients taking responsibility/ownership for their own health	
		Patient compliance and self-monitoring methods
	Diabetes awareness raising programmes	
Lifestyle	Newly emerging trends towards exercising	Cultural and social attitudes
	Availability of exercise facilities	

### Resources

The health professional focus groups had a recurring theme of describing the currently available resources and how they impacted on their performance and patients’ care. These included resources related to premises, staff and equipment. Their main observation was how these resources could be developed to enable them to become more available and accessible for their patients.

### Facilitators

#### Qualified experienced staff teams

Discussions in both groups highlighted the presence of strong teams of qualified members with complementary functions of care for patients with type 2 diabetes. Most members spoke about coherence and collective experience as a key strength for being able to educate their patients and overcome some barriers such as time limitations and overcrowding of appointments. Also, most had robust educational qualifications. The following quotes from the second focus group show how the team members complement each other, with a highly qualified doctor leading this team.

*Sometimes the clinics are overcrowded*, *with 20 / 25 patients*, *so we can go over with them the basics only*. *The nutrition clinic also helps us*, *so if I want to talk about a treatment*, *I transfer the patient to the nutrition clinic*, *as well as the diabetes education clinic*. *For example*, *if I wrote injection and new meals*, *I send the patient to the education specialists who help the patients to understand the part which I did not have the time to explain to them at the clinic*. (FG2-FD)

*We as nurses can help the patients referred to us by doctors by checking out their accumulative or fasting diabetes level; of course we would do this as per the doctor’s instructions*. (FG2-FNR)

*I as a nutrition specialist*, *write the notes*, *also the educator writes notes*. *These notes*, *which we take*, *help the doctor to identify the type of medicine and its quantity*. (FG1-MNR)

#### Educating patients through different communication channels

Good communication channels were seen as a way to strengthen health professionals’ educational messages to patients, avoid follow-up gaps and to be available for advice. Health professionals described making use of the already available communication channels that enabled them to talk further with their patients, which included social space in the premises, phone calls outside working hours and social media. This was also considered suitable for the local community culture as some health professionals felt that patients, for example, may prefer to receive messages via phone as a mean of communication.

One doctor referred to his use of twitter, and both he and one other referred to their use of Whatsapp.

*One video clip via WhatsApp to my patients is sometimes more effective than the educational sessions*, *I mean that the traditional methods are no longer that useful*. *The video clip is sent to the patient while being at home from a reliable source*, *and they can repeat it over and over*. *Sometimes they share it*. *I wish if there was more educational material that can be shared via WhatsApp*, *Twitter*, *Snap and the likes*. (FG1-MD)

*There is a patient with Dialysis and Diabetes*, *so I had to communicate with her via WhatsApp in order to observe her condition with her*. *She used to brief me about the rising level of diabetes till I could control it to reach only 300* [the nutritionist is likely referring here to high random blood glucose, normally 160-200mg/dl and above 300 mg/dl represents high risk], *so I have to follow up these critical cases*. (FG2-FNT)

### Barriers

#### High number of patients

Patients with type 2 diabetes attend one or two main specialist centres in their region. Due to the high prevalence of type 2 diabetes, health professionals were conscious of the constant pressure on themselves and the limited time they could spend with their patient to provide adequate educational advice about diabetes and how to manage it, especially for newly diagnosed patients. One Doctor, for example, described how she could not get the time to hear about her patients’ needs, questions or concerns due to the challenge posed by the high number of patients. The same issue was echoed by others:

*Instead of checking 20 patients*, *we'd be better to see 15 or 13 patients only so that we have enough time to sit with the patient and hear about his needs and concerns if he needed to enquire about anything… this way*, *he would take the right time if the booking was not too much*. (FG2-FD)

*Honestly*, *we need a long time with the patient*, *at least 20 minutes to explain what the nutrition is and how it could be adjusted*, *as well as what is the relationship between nutrition and diabetes in the first place*? *How can he*, *through proper nutrition*, *control the sugar in his body*? *What is the relationship between weight gain and high blood sugar*? *We need more time with the patient*, *but as the number of patients increases we cannot cover all topics*, *so we are forced to give the patients frequent dates for short appointments in order to be able to complete* [rather than one long session], *until we feel that the patient has received a good or acceptable level of education*. (FG1-MNT)

Health professionals across different backgrounds agreed that the number of staff, compared to the number of visiting patients per day was not sufficient enough to educate them to self-manage their type 2 diabetes and that improving the staff ratios could help to solve the problem. They highlighted that high numbers of patients resulted in not enough time to undertake high quality diabetes education with their patients.

*Of course the solution is possible by adding more staff as increasing the number of the staff already solves the problem of long waiting times for the patient till his appointment*. (FG1-FHE)

*As for this centre*, *we may increase the capacity within the centre by increasing the number of staff at the clinics*, *and we may increase the number of diabetes centres in the area*, *in general*. (FG1-MD)

#### Health centre location

According to health professionals, the type of patient education and care needed for patients with type 2 diabetes cannot be provided in the primary care clinics because they do not have sufficient qualified staff. This is only available in the secondary care specialised centres in the cities. This subsequently puts pressure on patients who have to travel from rural areas, while also resulting in increased capacity pressures on these centres:

*This is the only diabetes centre in Qassim*, *so the people come from every village such as*: *Al Dawadmi Village*, *so when the diabetes centres increase in this area*, *the pressure would be less upon us*. *There are diabetes centres in Onaiza and Buriydah*, *but if there is more than one centre*, *it may also decrease the pressure we face*. *As for this centre*, *we may increase the capacity within the centre by increasing the number of staff at the clinics*, *and we may increase the number of diabetes centres in the area*, *in general*. (FG1-MD)

*I agree with the doctor in covering more areas*, *and the patients who were examined and their analysis proved to be good*, *should be sent back to the hospitals*, *in order to decrease the pressure here*. (FG1-FNR2)

#### Strategy for patient follow-up

Health professionals seemed to be using their own initiative sometimes to reduce one appointment time for the benefit of another; but eventually patients’ waiting times are often extended or the time between follow-up appointments is protracted, which adversely impact patients’ compliance, among other issues.

*I mean that sometimes the patient as a start*, *especially at the beginning of diagnosis*, *may need less time*, *but sometimes the option is not originally up to the doctor*, *as he* [the doctor] *is committed to the appointment schedule and can do little to adjust according to the patient’s need*. (FG1-MNT)

*Sometimes the patient comes to the appointment after a period of time long enough for him to forget what I told him in the previous appointment*, *even if I gave him a paper or something to help him*, *he is unwilling to read*. (FG1-FHE)

#### Financial constraints

Although health care is provided free of charge in Saudi Arabia; there were some hidden costs incurred by patients. The discussions by the health professionals revealed that patients were sometimes unable to do physical exercise or self-monitor their condition because of the financial burden.

*I have a comment on the doctor's talk about the support matter*. *There are many patients who suffer from the cost; the ministry did not provide everything till now*, *so when I ask the patient to undergo an analysis he says*: *it costs too much and I can't afford it*. *Moreover*, *most of them do not have a Diabetes Analysis Device*, *they can't afford buying it*, *hence*, *it will affect them as well*. (FG1-FHE)

*But some patients are unable to go to the health club*, *for examples they do not have subscription* [reduced fee membership] *or the financial ability to go there*, *to be honest*, *most patients*. (FG2-FNT)

### Familiarity with self-management education programmes

Health care professionals seemed to be aware of the importance of self-management education. Most were working on educating their patients and raising awareness among them. However, their efforts were intuitive and the approaches used were variable among different professionals. The participants in this study had not seen a structured self-management education programme implemented as part of type 2 diabetes management.

### Facilitators

#### One to one tailored appointments for patient education

Most patients were seen at the beginning of their diagnosis by different professionals and were likely to benefit from health educator advice about nutrition and monitoring glucose level. Doctors also provided them with an explanation of the nature of their condition and how to ensure a good prognosis.

*The patient in his first visit must come to us in order to draw the broad lines and make him know the relationship between nutrition and diabetes*. *As I told you*, [nutritionists use “patient-tailored strategies”] *it is according to the condition of the patient*, *if he has any problems in terms of weight or anything else*, *we follow him up on a monthly basis on official appointments*. (FG1-MNT)

*We* [health-educators] *usually follow-on from the doctor’s instructions to the patient*. *However*, *some patients come here for consultations* [without being referred by the doctor], *so I determine my own appointments on which I can teach them how to monitor their blood sugar and how and when to take their prescribed dosages*. (FG2-FHE)

#### Patients taking responsibility/ownership for their own health

According to the health care professionals, some of their patients engaged with self-monitoring activities and wrote down notes on their condition to discuss with the professionals in order to help them improve their glycaemic control:

*By the diagnosis*, *often*, *the patient is given a table*, *a sheet of paper in which the analysis is written*, *its time and day*, *and he writes me the analysis and comes with it on the next visit*. (FG1-FHE)

*Communicate via text so that I can know about his condition*, *especially if he is a fresh diabetic or is taking medicine*, *if there is injection or anything new for him*. (FG2-FHE)

*Of course there are some patients*, *especially the young people; keen on following the instructions*, *perhaps it is because the education and thinking are different from older people's*, *while the older people are somehow difficult to be convinced because you want to change their lifestyle to which they are accustomed*, *so they may take some time to be persuaded*. *Those patients*, *who are 30*, *40 to 50 years*, *are very responsive*, *their analysis is getting better and the doctors feel satisfied with them as they follow the directions*. (FG2-FNT)

#### Diabetes awareness raising programmes

Although self-management programmes have not been fully applied, in Qassim, the professionals were used to some awareness programmes where they contacted the patients and the general public to provide crucial information about type 2 diabetes and how to manage it. Self-management education programmes were applied to a limited extent but did not follow a specific curriculum:

*As I said*, *the programme* [list of instructions from Saudi Ministry of Health to help patients to self-manage their condition] *was not applied in full; we tried to apply some aspects of it* (FG1-MD)

*Other than the doctors*, *there are for example the awareness programmes*. *We go to many places and Malls; there should be something like Diabetes National Day*. *There are education sessions with doctors*, *what if the patients attend those sessions and cared about it*. *Moreover*, *what if we have flyer*, *when they ask us*, *we answer; I mean to have a reliable source*, *not any source*, *it should be a reliable one*, *such as the awareness programmes*. (FG2-FD)

*We already created groups* [on social media] *such as ‘Insulin bump’; there is a new nutrition name ‘carbohydrates for diabetics’*, *in which there is the doctor and the educator along with the patients in order that if any patient asked any question regarding nutrition*, *I join the group and answer; if there is a question related to the medications*, *the doctor would reply*. (FG2-FHE)

### Barriers

#### Lack of previous systematic application

The mainline of discussion among professionals revolved around the lack of self-management education programmes such as DESMOND or DSME being used in practice or even being piloted in their centre, which serves a large province and provides health care for thousands of patients with type 2 diabetes:

*I personally know nothing about them*. [i.e. in his reply to if systematic self-management programmes have previously been applied]. (FG1-MNT)

*As I said before*, *I do not know about the programme in Britain and America*. *Of course an initiative like a programme would be the right thing*, *but it was never applied here in Qassim*. (FG1-MD)

#### Patient compliance and self-monitoring methods

Patients were likely left to their own initiative, readiness or willingness to decide how they would monitor their condition. Some patients would wait until their next visit; but others do not show up for their appointments. The same applies on following instructions or advice given by the health professionals:

*Sometimes*, *he is not educated or not well educated*, *meaning he is ignorant* [of diabetes and its complications], *so he needs someone to teach him in order to be more disciplined*. *Many patients are not interested* [not compliant], *not due to their ignorance*, *but they do not know yet know how important it is to get disciplined*. *Moreover*, *sometimes the patient comes to the appointment set by the doctor*, *which is so far*, *so he has to come after a long period of time but he forgets what I told him*, *even if I gave him a paper or something to help him*, *he is unwilling to read*. (FG1-FHE)

*Some of them follow the instruction and others don't*. (FG1-FHE)

*There are some patients who can't follow the programme as it is not suitable for them*. (FG1-FHE)

*I think that some patients prefer the easy things*, *they do not like sitting with other persons to educate them; they just want to serve themselves*. (FG1-FHE)

*When you sit with diabetics*, *they all listen to some of their methods*, *sugar control*, *and attend to some of their experiences*, *which* [from their point of view] *is better than the information or more influential than the information that the doctor mentions*. (FG1-FHE)

The professionals were keen to see and follow-up their patients. However, the booking system impacted their ability to control how frequently and when they could see a particular patient. As such, some patients were seen frequently enough, others perhaps too often, while there were patients who did not get the chance to see all the members of the team.

*Sometimes they are educated by the doctor if he has time*, *and other times they are not*. (FG1-FHE)

*When there is too much booking*, *some patients feel stressed and the time is not enough*. *Sometimes*, *if the time is not enough*, *I feel that I did not give the patients all the information he needs and give him an appointment for the next day; hence the booking increases to 20 or 27*, *so I give him an extra appointment in order to complete the things he needs*. (FG2-FHE)

*Well*, *for the same patient*, *I may set an appointment according the condition of the patient*, *some of them come on a three months basis and others come every six months*, *also there are some patients who come for consultation at any time*. (FG2-FD)

### Lifestyle

The professionals discussed their patients’ lifestyle and how it could affect their compliance with self-management programmes. They gave some positive and negative examples, including the changes with current trends in society.

### Facilitators

#### Newly emerging trends towards exercising

According to the health professionals, some Saudis are keen to move around and practice more physical activity, especially the younger generations. A growing interest in practicing sports was evident.

*The culture of walking spread more than before*, *as well as the bodybuilding*. *Hence*, *culture is subject to change and people are satisfied with this thing*, *but it needs some kind of support and motivation*. (FG1-MD)

*I recently read on Twitter that there was a walkway in the housing area*, *in which there were groups walking*. (FG2-FD)

#### Availability of exercise facilities

Health professionals talked about new residential areas that had been designed to provide walkways and fitness clubs were also available. There were also conversations in the focus groups about golf courses and swimming pools.

*The activity classes can be increased*, *and for example*, *the role of fitness can be adopted; the tracks are now common*. (FG1-MD)

*I think that* [physical activity levels] *can be made in sports clubs for example*, *which contains everything such as walkways*, *tracks*. (FG2-FHE)

### Barriers

#### Cultural and social attitudes

According to the professionals, overweight and obese patients were frequently encountered in their type 2 diabetes clinics. They believed that this was influenced by dietary habits and traditional food.

*The main problem we have in the Kingdom of Saudi Arabia is the food style or*, *in general*, *the lifestyle*, *which is not to practice exercises enough*, *and also to rely on the qualities of high-calorie foods*, *high amounts of fat and also high amounts of sugar*. *I think that anything needs a government programme to change it*, *needs to have a huge programme to try to change the lifestyle of the community*. (FG1-MNT)

For some professionals, they felt that they had to work hard on building trust with their patients. Some patients believed that traditional recipes or medications could provide them with better and safer solutions that could not be matched by modern medicine:

*Some patients come here while already convinced with their own ideas*, *whether traditional medicine like what I said or they are already convinced*. *He may come to the clinic as a duty that must be done*, *I mean that he is already convinced*, *and this is a difficult matter*, *not only the elders*, *but also other persons*. (FG1-FHE)

In Saudi Arabia, people do not simply go out of their homes and walk. Professionals believed that this would require planning, motivation and organisation. On the other hand, females still felt restricted by when, where and how they would go out for a walk.

*Because of the culture*, *no one would go out to walk while wearing the home dress*. (FG2-FHE)

*I suggest providing a closed building*, *such as the sports club*, *for the female patients*, *in which they can do every sport in such club*, *such as swimming*, *walking or other things*, *provided to be in a closed place*, *but if the marathon was made in an open area*, *I think they won't come*, *even if it was the Health Day*. (FG2-FD)

A further concern for some professionals was the culture of people having a reliance on travelling everywhere by car, representing a further limitation to routine daily physical activity:

*I mean we should limit using cars because people today go to the mosque by car*, *or to the greengrocer's* (FG1-MD)

In summary, the themes that emerged from both focus groups were consistent and the participants shared similar views on different topics. They agreed on pressures related to the availability of resources. Participants were willing to learn a systematic programme that educates self-management; however, they had not experienced one before. They also felt the need for such programmes and believed that they were applying parts of them but without clear guidance or structure. Older patients required special considerations to be taught new concepts and participants felt that the culture had some barriers especially for exercising in public places; traditions and customs were strong catalysts and suppressors alike; they need to be considered within any cultural adaptation for self-management education.

### Patient interviews

Twelve (out of 31 approached) patients with type 2 diabetes participated in a one to one interview (S1 Appendix). Six patients were males (mean age 47 (38–56) years) and six patients were females (mean age 44.7 (25–56) years). Table 1 shows the participant identifier for each patient.

Three main themes emerged (Table 3), again presented as facilitators and barriers to diabetes self-management. Newly acquired habits (mainly post diagnosis) were identified as a facilitator, and cultural barriers and bad habits (mainly pre diagnosis) were identified as barriers.

**Table 3:** Patients’ interviews themes and subthemes.

	Subthemes	
Theme	Facilitators	Barriers
Habits	Healthy diet	Unhealthy habits
	Active lifestyle	Unwillingness to practice sport or physical exercise
	Monitoring blood glucose	
Health education	Receiving education at health centres	
	Self-taught awareness diabetes awareness	
Culture and society		Social restrictions and attitudes

### Habits

This theme reflects post diagnosis changes in patients’ attitudes and behaviours towards diet and physical activity.

### Facilitators

#### Healthy diet

Many patients expressed their interest and/or commitment to eating healthier food and their observance of what, when and how much they eat.

*Yeah*. *I always eat leafy vegetables as they don’t raise the diabetes*. (FP-1)

*I’m on the diet they* [Health centre] *told me about*. *They say I should have one piece of fruit per day*. (FP-1)

*I keep eating the fruits and vegetables continuously because the doctor advised me to do that*. *The doctor advised me to eat the fruits which don't contain more sugar*. (FP-2)

*I was decreasing the calories I had as well as sugars and fats*. (MP-3)

#### Active lifestyle

Patients also talked about their commitment to a healthier lifestyle after being diagnosed with type 2 diabetes. A relatively high number of the patients described becoming more active.

*I walk every day*. (FP-1)

*When I practice sport especially walking*, *this is reflected in my psychological state*. *So*, *I am keen on practicing it continuously*. (MP-1)

#### Monitoring blood glucose

Some of the patients reported carefully monitoring their blood glucose level, and their awareness of its importance:

*Yes*, *of course I have a detector and I follow up the analysis when fasting and after the main dishes*, *even the random test*, *sometimes I do it*. *In regards to the advice*, *I resort to the physician of the centre in the district periodically asking him for advice*. *Regarding the medicine*, *I have them regularly*. (MP-2)

*She reported the analysis for me and found that proportion of glucose was 50*. *She said that it was low*. *She brought dates and juice for me*, *and I was able to control it and I thanked Allah*. (FP-2)

*I did a test at home and found a simple percentage*, *so I visited the clinic in order to undergo a complete analysis when they told me that the sugar level in the blood is high*. (FP-3)

### Barriers

Bad habits before diagnosis’ captures patients’ behaviours before they received a diagnosis of type 2 diabetes: their negative behaviours towards diet, physical activity and lifestyle. Some patients managed to stop these habits and others were feeling the need for amending such behaviours after being diagnosed with diabetes, but there were some participants, who seemed indifferent.

#### Unhealthy habits

A few patients reported smoking and how they were finding it difficult to quit or adjust after being diagnosed with type 2 diabetes.

*I never took the subject of smoking seriously until now*. (MP-1)

*Unfortunately*, *I am a smoker* (MP-5)

There were some reports of patients’ avoidance of fruits and vegetables. Old bad eating habits, which included eating fatty or fast food or not being keen on fruit or vegetable consumption.

*My relation with fruits and vegetables was not so good really especially the fruits*, *Is this a food routine to which I’m used to*?!! *Is this a social behaviour*?!! *I do not know but my relation with the fruits is still very superficial*. (MP-1)

*Not much*, *but I do eat them* [fruit and vegetables] *now*, *but I do not show great interest in them* (FP-6)

There was moderate frequency of patients’ reports of earlier incautious food consumption. Some reported slipping back to these dietary habits. This can be seen associated with less awareness of the negative impact that this had on their health.

*Yes*, *I was eating fast food but now sometimes not usually*. (MP-2)

*Quickly I returned to the natural situation and I began to eat everything like sweets and all items*. (MP-3)

*Nevertheless*, *unfortunately I know many people whose diabetes levels are 400 and 500*. *A huge mistake*. *Why*? *If it is 200 and below*, *that is acceptable*, *but 300*, *400 and 500 are the levels of those who do not take care of anything*, *are not interested in their treatment*, *nor are interested in eating healthy food*. *They only harm themselves*. *I mean*, *if this year everything goes well with them*, *they would eat openly*. *However*, *that will not last*. *Two or three years from now*, *they will find themselves complaining about all their body organs*. (MP-5)

*I mean I crave something and eat it knowing it is harmful*, *but it is something I desire*, *I cannot control it*. (FP-6)

#### Unwillingness to practice sport or physical exercise

Patients often reported their previous tendency to avoid physical activity. Some patients attempted justifying their sedentary lifestyle.

*When I suffered from diabetes*, *the doctors advised me to walk*. *I didn't used to walk at all and I didn't try*. *So*, *my mistake is that I didn't try to walk*. (FP-2)

*Walking is sport*. *They never said to me anything related but they told me about another sport for which I shall prepare myself; they* [health professionals] *never do anything for me*. (FP-2)

*I swear*, *there is no doubt that due to being overweight*, *this affects me as I suffer an attack if the weather is not pure… I cannot walk*. (MP-3)

### Health education

This theme was built on the self-reported awareness of type 2 diabetes, how it could be managed and how the patients developed such awareness. However, no barriers were reported that hindered their health education or awareness.

### Facilitators

#### Receiving education at health centres

Almost all patients talked about being educated on their current health condition. However, the level of such education, or their perceived judgment on the level and quality, was variable:

*The* [main] *source* [for education] *is the diabetes centre*, *I do not believe in anything else*. (MP-6)

*It is according to the diabetes level*. *I follow the instructions of the doctor*. *My treatment is regular and thank God my condition is stable*. (MP-5)

*I have got some of these sources from the nutrition clinic at Diabetes Centre here*. (MP-2)

#### Self-taught awareness

Some patients’ reported on their self-developed knowledge of type 2 diabetes.

*I have many sources that I got and I try to read anything about diabetes*. *There are always lectures and seminars about diabetes and I attend them continuously*. (MP-3)

*They* [health centre] *are the ones who teach us and tell us whatever we want to learn*. *I know people at home who know about the disease and they teach me*. (FP-1)

### Culture and society

Unfortunately, patients did not give a sense of cultural or social support, and some implicated a barrier created by some social practices or conventions.

### Barriers

#### Social restrictions and attitudes

Patients discussed how cultural barriers can interfere with their self-management or self-management education. Only two patients reported feelings of being made to feel guilt or blame for developing type 2 diabetes. However, they found it difficult at times to pass a food offer, which was not suitable to their condition. The patients explained that this may have led them to mismanage their condition.

*We need more support*. *I can tell you that the Saudi people have many events and occasions which contain eating food*. *The life became hard*. [i.e. referring to the pressure of social “eating”, and the negative view for not joining in] (FP-2)

Patients also raised the issue of how the community had some misconceptions about practicing sports, or who should be practicing sports. On the other hand, the places where sports could be practiced, including streets and playground were considered insufficient or not accommodating enough as reported by one female patient:

*Nowadays there are no tracks in the clubs for they* [i.e. the males] *want them only for themselves*. (FP-6)

Financial constraints to taking part in physical activity was also noted by one patient:

*We would like to*, *but everything here costs money*, *do you understand*? *We would like to walk and to go to the club*, *but everything is in return for money*, *we have not enough money*. (FP-4)

## Discussion

The current study found that health professional participants had no prior knowledge of programmes such as DESMOND or any other similar programmes. However they attempted to create their own system for educating their patients, when they had the time. All patients from or outside the city were managed in the centre as they could not receive the care at their local primary health clinics. It was clear in the focus groups that there was mounting pressure on staff to treat a daily high number of patients. However, both patients and health professionals recognise the importance of good equipment standards, staff qualifications and experience in such specialised endocrinology centres. Outdoor physical activities are constrained by social frames, especially for women and due to dependence on vehicles; however indoor sport facilities are available, but some health professionals raised affordability as a concern. Although traditional food is almost a must in social gatherings, there are some emerging trends towards healthier diet.

While self-management programmes such as DSME and DESMOND have the common aim of educating patients to acquire the key skills to become independent managers of their own condition in order to maintain a better quality of health and life, the UK based DESMOND programme is applied on a national scale through the national health service (NHS), which is more similar to the health system in Saudi Arabia [12, 30, 31]. However, this study, which took place in the only endocrinology centre in the Qassim provenance, shows that previously trialled or piloted self-management education programmes in Saudi Arabia [4] had not been disseminated or discussed at least within this part of the country, which is the fourth populated area in Saudi Arabia [4]. When taking into consideration that patients with diabetes can only be seen and managed in such centre, it is likely that health professionals are well placed for describing the pressure on them, and their patients, during the appointments. The main aim of DESMOND is to use standard methods for training diabetes self-management educators a systematic approach by which they educate type 2 diabetes patients. This standard approach follows the guidelines of the National Institute for Health and Care Excellence (NICE) [31]. It is hard not to notice that this is quite fitting as solution of the high volume of patients. If educators become available in primary health clinics then many of these patients will not need to travel to the specialised centres. Moreover, if the patients are properly educated to self-manage their diabetes, then their need to be seen by a specialist will become less, due to less complications and better ability to monitor their condition [32].

Cross-sectional studies in different geographical populations in Saudi Arabia showed that many communities are keen on traditional food [33, 34], which is high in calorie and rich in fat and carbohydrates [33]. This was also associated with obesity or being overweight [33], mainly among females [34]. According to these studies, the problem is escalated by limited physical activity and sedentary lifestyle [35, 36]. The views expressed by the patients and the health professionals in our study gives support to these findings. However, they also show that there are emerging trends and desires for an improved lifestyle. It is possible that people in Saudi Arabia are becoming more aware of healthy diet paradigms and the importance of being active. Nevertheless, these views were expressed by individuals, who were already diagnosed with type 2 diabetes, and it should be noted that some did not adopt a healthier lifestyle until they were diagnosed. Also, the findings in this study found that cultural barriers restricted females from exercising and participants often used a car as their main form of transport, regardless of distance to their destination. As noted by some health professional and patient participants, females are limited with regard to accessing physical activity facilities, likely due to the conservative traditions of the society. In a study that reviewed most of the initiatives that aimed to encourage the Saudi community to practice further activities, it was found that most of these initiatives were sporadic or short-lived and had a limited impact, especially for women, including young girls [36]. In our study, for both health professionals and patients, it emerged that there were community or self-imposed restrictions on practising outdoor sport. It is unusual for Saudis to go out for walks, whether long or short. However, for females, the social barrier represents a bigger challenge. Nevertheless, the discussions showed that most patients were motivated to do more to get themselves fitter, but they required proper guidance and improved community awareness about the necessity of frequent activity in general, for both males and females, and for them as patients with type 2 diabetes in particular. A key aspect for a DESMOND educator is to be able to tailor their approach according to the patient experience and history, whether ongoing or newly diagnosed [31]. For DESMOND to be successful in Saudi Arabia, the disseminated standard will need to take into consideration particular needs, which are shown in this study, such availability of the sports facilities nearby their patient, the cost involved and occasions associated with traditional food. In other words, they will need themselves to be educated about the local environment and geography in order to be able to provide an efficient education to their patients [31].

The high number of patients, lack and centralisation of specialist centres and specialised staff restricts the opportunity to educate patients with type 2 diabetes on how to self-manage their condition and achieve better control of their diabetes. This is aggravated with the absence of national self-management education programmes. On the other hand, community traditional concepts can interfere with patients’ compliance and ability to adhere to health care professionals’ advice. Social studies report the remaining influence of the family, or the community as a whole, on its members. This influence can delay, alter or impact individual’s health seeking attitudes [37]. Type 2 diabetes self-management education programmes need to be open to the community, not only to help the patients with type 2 diabetes, but to also help reducing the number of people who develop type 2 diabetes. This will help to break the aforementioned cycle of service limitations caused by the high number of patients. There is a need to train staff to apply a systematic approach towards educating their patients, which can also be tailored to individual needs. Nevertheless, both staff and patients are likely to welcome self-management education programmes, especially if they are more suited to the community by providing, for example, advice on how and where they can do more physical exercise and how their families can support their self-management goals, such as observing dietary intake. Also, the use of an attractive medium for communication such as digital health interventions (via apps), social media and videos has the potential to save patients travelling to distant health centres. This could benefit the Saudi community, which is quite engaged with smart screens and may also free up some time within clinics for patients who do require an appointment with a clinician in person. As mentioned previously, the MOH has recently provided its patient care telecommunication hub services. This hub could be a suitable medium for introducing electronic diabetes self-management programmes, to enable qualified educators and trainers to reach the largest possible number of the population, including those with limited mobility. The telecommunication services can be utilised to advertise and raise awareness of such a programme or particular aspects of it, such as public health messages about diet and exercise to the whole community, which could provide one to one confidential communication between the health professionals and patients under their care.

Limitations of the study include it being carried out in one locality, participants views were not re-examined by means of a lateral study to see if a different group of participants would concur with the themes and subthemes identified here. It is not possible to exclude bias or favouritism among participants in such convenience sample, who may intrinsically support additional resources or care plans. Strengths include the in-depth interviews and focus groups with a range of experienced health professionals and patients, including both males and females.

The findings from this study, within their limited scale, provide grounds for a Saudi specific self-management programme for type 2 diabetes. According to the Kumpfer’s cultural adaptation model, the above-mentioned findings can be used to make some of the adaptations to a self-management programme that could be piloted within the study locality to be robustly tested for its effectiveness. The findings also reflect supportive views of key stakeholders, namely the patients and the health professionals responsible for their care, of a self-management programme and highlights gaps and readiness in society, patients and the health care system for type 2 diabetes care. Such a programme should help to alleviate many of the challenges that are currently facing diabetes care provision in Saudi Arabia and help to tackle the rising prevalence of type 2 diabetes in the country.

## Supporting information

S1 File (DOCX)

S2 File (DOCX)

S3 File (DOCX)

S4 File (DOCX)

S5 File (DOCX)

S6 File (DOCX)

S7 File (DOCX)

S8 File (DOCX)

S9 File (DOCX)

S10 File (DOCX)

S11 File (DOCX)

S12 File (DOCX)

S13 File (DOCX)

S1 Appendix (DOCX)
