# Supplementary material for: Cultural adaptation of self-management of type 2 diabetes in Saudi Arabia (qualitative study)
Source: PLoS One. 2020 Jul 28;15(7):e0232904. doi: 10.1371/journal.pone.0232904 (PMC7386581; doi:10.1371/journal.pone.0232904)
Supplement: S1 File — (DOCX) [file pone.0232904.s001.docx]

Focus Group 1

Doctor: For us, every day we see patients, it is supposed to see them for three to four months, supposedly. In reality, on the contrary, we can only see them every 6 months and more than this, regardless of the importance of the visit to the patient. Sometimes, I bring them as "Over booking", on an appointment, but I tell them to come in a given day.

I know that he needs a near visit, so I add him on my list. We suffer from this situation, as it is supposed to be from three to four months, but in reality it is six months. Sometimes, the patient who I need to see, I mean his medical condition that needs so and so, I bring him as "Over booking".

Doctor: Well, we are organizing time here, in the clinic for example, I have to sit with the patient, if he is a new patient, from 15 to 20 minutes at the clinic. It is possible, according to the law, to see 20 patients, and if the time is distributed, I would not be able to see them all, on one hand; on the other, if there are a staff, i.e. more persons, they will see more patients, may be less or more. Therefore, the clinics would reduce the waiting time, this is what I suggest.

Nutritionist: Depending on the situation. I mean that sometimes the patient as a start, especially at the beginning of diagnosis, may need less time, but sometimes the option is not originally up to the doctor, as he is committed to the appointment schedule and can do nothing at his option and we cannot interfere as well.

Diabetes educator: Of course the solution is possible by adding more staff as increasing the number of the staff already solves the problem of waiting time for the patient till his appointment.

Doctor: In addition, this is the only diabetes centre in Qassim, so the people come from every village such as: Ghareeb Al Dawadmi Village, so when the diabetes centres increase in this area, the pressure would be less upon us. There are diabetes centres in Onaiza and Buraidah, but if there are more than one centre, it may also decrease the pressure we face. As for this centre, we may increase the Capacity within the centre by increasing the number of staff at the clinics, and we may increase the number of diabetes centres in the area, in general.

Nurse 1: I agree with that.

Nurse 2: I completely agree with that.

Nurse 1: Yes, more clinics should be opened and more doctors should be brought to the centre here in order to decrease the number of patients and be distributed to the clinics.

Nurse 1: Yes, and decrease the time period from 6 months to 3 or 4 months as the doctor said. Moreover, the booking list of every doctor should have less number of patients in order to give each patient his due time.

Nurse 2: No, I agree with the doctor in covering more areas, and the patients who were examined and their analysis proved to be good, should be sent back to the hospitals, in order to decrease the pressure here.

Nurse 2: Yes, Health Centres.

Nurse 2: I completely agree with what the doctor stated.

Diabetes educator: No, 4 months would be enough in order to be able to see the cumulative every three months.

Doctor: Well, I try to make an effort with the patient, but I am surprised that time is pressing me, so we began to do a consulting course for dialectologists who are being in the centre now, and giving us a strong boost. For example, how to increase the dosage? Where and how to make the injection? These matters used to take more time when I give the insulin for the first time.

Back to the question regarding the role of Primary health Care, supporting it with at least a number of Family Medicine Specialists and over, this will help in decreasing the pressure, and it is possible to do that, and hence transferring the uncontrolled special cases from type 1 that have problems and need Insulin Bump and the alike.

Diabetes educator: Praise be to Allah, it is enough as educated in diabetes, and the number of patients I receive every day. Sometimes, there is more pressure from the clinics, especially the clinics of the first type of diabetes, which have some pressure, but often the time is enough.

Diabetes educator: Yes, the patient takes its full time and more, it is possible to have some pressure in certain days when the patients are too many, so many times I have to shorten the time taken with the patient in order to give the next one his right time as well.

Diabetes educator: Not all of them, some of them follow the instruction and others don't.

Diabetes educator:: Sometimes, he is not educated or not well educated, i.e. he is ignorant about such thing, so he needs someone to teach him in order to be more disciplined. Many patients are not interested, not due to their ignorance, but they do not know yet the matter to get disciplined. Moreover, sometimes the patient come on the appointment set by the doctor, which is so far, so he has to come after a long period of time but he forgets what I told him, even if I gave him a paper or something to help him, he is unwilling to read.

Diabetes educator: Do you mean to educate them?

Diabetes educator: Sometimes they are educated by the doctor if he has time, and other times they are not.

Nurse 1: Yes, it is enough.

Nurse 1: Yes, it is enough.

Nurse 1: No, it is enough for Type 1, but Type 2 needs more time in terms of education, treatment, its method and everything explained to him.

Nurse 2: Because the Type 1 is somehow difficult, they are children and cannot control themselves.

Nutritionist: Honestly, we need a long time with the patient, at least 20 minutes to explain what the nutrition is and how could be done, as well as what is the relationship between nutrition and diabetes in the first place? How can he, through proper nutrition, control the sugar in his body? What is the relationship between weight gain and high blood sugar? Time is long and our need for time is important and present, but as the number of patients increases we cannot speak in all things, so we are forced to give set to the patients close dates in order to be able to complete, and to convey the idea and feel that the patient has received a good or acceptable awareness.

Nutritionist: Frankly, there is a percentage that has insight and awareness, so we get results, on the other hand, there are patients who are not interested, so their condition is the same as they came in the first visit. The problem is that there are patients who rely on the treatment of diabetes through the popular things and tips spread throughout the Internet, and come to the diabetes center here just to take the treatment of pressure.

Nutritionist: Well, the problem is in the awareness of the community as a whole. The first thing is the Mass Media, there is no control on them in our country; any one may appear in the TV Shows and talk about any matter, there is no regulation, so you find many people talking about all topics, especially in the social media, he may even recommend a treatment for the diabetes for example, and give prescriptions and diets despite having no knowledge in the health field in the first place, but any way, he is not being followed.

Doctor: I want to add something. One day I wrote a tweet on Twitter, only one video clip spread via WhatsApp to convey the flyer, whether true or false, to the community more than the educational sessions, the collection of patients and normal video clips; I mean that the traditional methods are no longer useful because they now believe the video clips shared via WhatsApp, why is that? Because the video clip is sent to him while being at home from a reliable source, and repeats it over and over. Sometimes they add a religious character to it such as: Share and got rewarded by Allah, and the alike. I wish if there was an educational material contained in the communications shared via WhatsApp, Twitter, Snap and the alike which activate such things.

Doctor: Yes, to be formal and from officials to transfer the desired image or the required flyer such as the doctors who should have a role in it for providing education or nutrition, under the umbrella of the ministry, and to publish formal flyers in the face of these trends and rumors. There should be persons skilled in education and any health programs they wish to apply on the community which should contain certified scientific materials through these persons only.

Doctor: Don't worry; I think they get the flyer, because he has a child, a brother or sister who plays the video clip for him along the day without reading or writing, only to watch and hear the video clip and gets the flyer you want. I think that they face no problem with that, but because of our experience in the centre with the people whom we educate and do not give them a new medicine, the first thing to do is to communicate with them with conviction.

Sometimes, I think it is right to add a dose of insulin, for example, but if he is not satisfied with my words originally, he will not submit, even if I gave him the medicine and sent him to education, the first thing is to have a simple discussion and agreement with the patient before sending him, on one hand, and if I classified them, you may find that 30 % of them are abide by these things and are aware of what I told them.

The rest of them, 50 %, may forgets half what I said to them, and they came on the next visit without any change. I see futility, which means that the effort made is not equivalent to the resulting effect.

Diabetes educator: I support the doctor in what he said that the patient absorbs a small part and forget the rest, so we have something in the clinic here called the Feedback in which we offer him something; I mean to work on a particular matter which eventually benefits him. I make him do what I said to him practically, through which I think it may help him to be settled in his head. Moreover, when the patients came to the centre for the second time, I ask them about what I told them in the previous visit and see whether they absorbed and applied these things or not; if they did not apply it, they would forget, which I measure also.

Diabetes educator: As the doctor said, some of them are unwilling to do that; so if the patient is not convinced with what you told him, he will not apply, we know that thing; if the patient is convinced with what I told him at the clinic, he would apply such things.

Diabetes educator: Yes, there are.

Diabetes educator:: May be the time; I mean the patient should see all of us, the doctor, the nurse, the health educator or the nutritionist, and we should all say the same thing to support each other in order to persuade him, I mean to have more than a person. Some patients attend only to the doctor, they see the diabetes educator or the nutritionist; if the doctor supported what I told them, or told them something and I completed, I feel that they are convinced.

Diabetes educator: We can give them Flyers or things like that by which they may be convinced.

Doctor: The family members for instance; a mother brings her daughter with her or a brother brings his own brother or sister; I mean that one family member with the patient to hear the instruction with him in order to convince him at home. Involving the family with him in the treatment plan is a great support for the application of medical advice, in addition to flyer and videos, or maybe he can search for himself as there are many educated persons who can read and get educated about his illness, the medicine prescribed and the side effects of such medicine, so that he can discuss with you in the next visit. This thing is there and I expect the involvement of the family which means a great support; you mean physical stimulation and moral stimulation?

Doctor: The encouragement may be provided through the results if he has a controlled level of diabetes or the side effects are decreased etc. This is physical stimulation, but we don't have such moral stimulation, except by saying: "Great, your diagnosis is very good" I mean to reassure the patient, otherwise we have nothing.

Diabetes educator: Some patients come here while already convinced with their own ideas, whether traditional medicine like what I said or they are already convinced. He may come to the clinic as duty that must be done, I mean that he is already convinced, and this is a difficult matter, not only the elders, but also other persons.

Diabetes educator: The glands in the traditional medicine.

Diabetes educator: Especially the glands as the glands are important for the traditional medicine; sometimes they abandon the treatment because of it, many of them.

Nurse 1: Frankly, the same as she said.

Nurse 1: I don't know.

Nutritionist: I think, as a simple imagine, that the solution is in two things, the first: the presence of a team similar to what is here in the primary care centres.

Doctor: The presence of a dietitian, educator for diabetes and a doctor related to Family Medicine to be exist so that there is a follow up and hence raising awareness of the patient. When the patient comes here, there should be planning for the plan of treatment. It is impossible, with such large number and as a specialized centre for a complete area, that they start from the very beginning and teach him what the diabetic is, trying to fight rumours instead of making a plan for the patient.

The second thing: The existence of a third party working on this subject, such as associations and charities that deal with diabetes, educate diabetes patients or educate the community as a whole. These specialized centres are supposed to be existed, although they are specialized scientific centres dealing with treatment, as well as practical development and practice. These charities can reach the patient in the streets, at homes or the shopping centres, and try to get him the new ideas, fight rumours and try to raise the level of awareness in the community; I think the solution is in these two things.

Doctor: Yes, I do.

Doctor: I can summarize the subject that our discussion here is about the Secondary profession; all the problems we face such as the lack of time, and lack of education of people and non-response, is all secondary, right? So we should work on the primary, to start from the base.

Doctor: Educating people through the mass media for the healthy community differs from those who suffers from diabetes, so they are the main support for you, as they would relief the efforts. In the beginning, an effort will be made at the community level, but at the end, the secondary things that we are suffering from will be alleviated.

Diabetes educator: When the patient visits the clinic, I set an appointment for him, orally not written, after one or two weeks on a definite day and at a definite hour. Sometimes I join the appointment with the doctor's, i.e. when he see the doctor, he may come to the clinic after one or two months, according to the patient.

Diabetes educator: By the diagnosis. Often, the patient is given a table, a sheet of paper in which the analysis is written, its time and day, and he writes me the analysis and comes with it on the next visit.

Doctor: As for the patient, I ask him about what he feels, and the things that I notice and the objective things in the analysis supported by his words about the symptoms of the diabetes, its level, thirst, drinking water, frequent eating and frequent urination, God bless you, how often he got up from sleeping, loss of weight and other similar things in addition to the public health.

These are the main things. There are also certain people whom I ask about the psychological side, and other people, from the age of 40 and more, may ask me about the sexual relations with their wives and other complaints, but generally how can I judge the patient? Based on analyzes, symptoms and side effects.

Nutritionist: For us, there are two ways: Depending on the patient's condition which need an appointment, such as a diabetic who has an overweight; so we give him the appointment and check nearly everything. We develop a monthly plan for him to notice the changes after every month. There are also some patients, if his weight is ideal but has problems in not controlling diabetes, so we set his appointment as per the doctor's because we need to have a look at the analysis, except in very simple cases if the patient wishes to review periodically, at our own appointment.

Nutritionist: Perhaps the Miss is diabetes educated, but we, as a feed clinic have our own appointment. However, type I patients on every visit to the doctor must visit us, as well as the newly diagnosed patient. The patient in his first visit is a fresh person who must come to us in order to draw the broad lines and make him knows the relationship between nutrition and diabetes.

These things are compulsory of course; whenever the patient came, he must come to us in our booking clinics. As I told you, it is according to the condition of the patient, if he has any problems in terms of weight or else, we follow him up on a monthly basis on official appointments.

Doctor: Mr. Ahmed handled a great point which is the type 1 patients must visit the nutrition clinic. I think that if I told them that they should visit the nutrition clinic, they will not go; so the patient comes to the doctor and the doctor sends him to the nutrition clinic, but he does not go; he only takes his medicines and just leaves. If you tried to convince patients or enforce them to review the nutrition clinic, it would be evidence that they don't see nutrition as a part of the treatment, it is clear. This is what I meant.

Nutritionist: Excuse me; there are also other things that help the doctor to develop the treatment plan, such as the Type 1 patients. The patient comes with a form in which I, as a nutrition specialist, write the notes, also the educator writes notes. These notes, which we take, help the doctor to identify the type of medicine and its quantity.

These things are so important for the doctor to be cared about, such as the types of food for the patient; some patients depends on two or three types of food only with no change, so we try to give him medicine suited to the types of food because we can't change the habits of the patient. Some patients find it difficult to change their habits, especially the Type 1.

These paper notes are clear to the doctor, helping him greatly to develop a therapeutic plan that helps the patient healthily.

Nurse 2: No. listen, there are patients do so by themselves, but the majority of them are being enforced, meaning that they must go to the nutrition specialist, the educator and then see the doctor.

Nurse 1: I have nothing in my mind now.

Diabetes educator: Yes.

Diabetes educator: Unfortunately, the patient does not need encouragement; he should know what he is doing. Some patients see the doctor only if they need treatment, and others visit us every day, so it is measured by the patient's awareness, meaning that we cannot say that even the first type, all of them need to be forced, but this kind of minds are exist as well as the interested people, this is reflected on their consciousness, and even reflected on their analyses, their diabetes is nearly controlled. We return to the basic problem of awareness and periodic follow-up, we can't carry out the two methods as specialized centres.

Doctor: For consciousness, firstly I always tell the patient this definition of their disease in Arabic which they must read about it, I give him, for example, a reliable web site on the Internet, a flyer or anything as I said, or to educate the family member simply. Secondly, mainly the mass media, for example, the Ministry of Information is responsible, Malls are responsible and charities are responsible. I don't put this thing only on one entity; there must be cooperation At the whole community level, to raise awareness. The Ministry of Education is possible to add things that have become apparent, such as sugar, stress and obesity, the activity classes can be increased, and for example, the role of fitness can be adopted; the tracks are now common.

The culture of walking spread more than before, as well as the bodybuilding. Hence, culture is subject to change and people are satisfied with this thing, but it need some kind of support and motivation, I mean we should limit using cars because people today go to the mosque by car, or to the greengrocer's. If he enters the daily things in his program, it can be a sport on its own. I repeat and say raising the awareness of the entire society with its full associations, not just health, I means the Ministry of Health.

Diabetes educator: We talk to the patients who come to us, and support my words with a flyer or a video. Firstly, I undertake an assessment and let them see it; what are the things that they love, hate or fear? to correct the things after that. We also have a role outside the center, where we are doing campaigns in malls, and also there are scientific conferences which have a big role in getting them aware.

Nurse 1: Like flyers.

Nurse 2: Encouraging the patient.

Nurse 2: I repeat the same words of the doctor to them again, distribute the pamphlets to them and encourage them to attend courses or places where we are, where we educate them.

Diabetes educator: I think that there are two ways: the most important way that anyone should deal with any position based on the culture he has. If a diabetic patient has no news about his illness, he will not be diagnosed. He doesn’t know any information on sugar, sugar hazards, sugar control methods or prevention, the time when did he get diabetic and diagnosed. So, if this education given to him is late, his condition will be difficult.

He will be looking for the easiest things to deal with, such as a treatment mix, or to control his food and practice a sport to control the diabetes. Targeting people, even the healthy ones, is important, especially those with diabetes, because I don't think that there is a house free from a diabetic, and the Saudi person's exposure to diabetes is high, especially if we build on the life style here and genetic needs.

Moreover, when we talk about a diabetic, the presence of groups of diabetics with each other and share the same problem, with different ways to deal with the same problem, this will create a different way of handling which is better than giving it easy information. When you sit with diabetics, they all listen to some of their methods, sugar control, and attend to some of their experiences, which is better than the information or more influential than the information that the doctor mentions.

Nutritionist: From my point of view, the diabetic needs something simple provided by hand, don't sit with them as they will not be persuaded, I need something more simple than this, I don't know.

Doctor: These groupings are exist and beneficial in many diseases, as for diabetics, why not? There were mental illnesses and physical diseases, for example, so the whole group shares the same disease and the same experiences, and the treatment method is universally effective and beneficial, and is one of the successful methods.

Doctor: I think the minister has put forward an initiative, which is about the groups sharing the same disease, to share experiences, it was sent it to us by e-mail less than a month ago; it exists but I do not know about its activation now.

Nutritionist: I think even if you did that, it is important to have a health facility as the centre of the neighbourhood, the presence of neighbours with each other and under the supervision of a specialist doctor is possible to deny any information mentioned by any one, you can't only gather them together and leave them because someone may come and cast an idea that you do not expect which may confuse everything, so I imagine that they should be under a controlled care.

I mean right things as if there's a specialist doctor, he can interfere to correct any information made by any of the participants. Moreover, the existence of an official establishment, the primary care centre in the neighbourhood, the presence of well-known persons and neighbours, would create an excellent environment as I think, and is also capable of being controlled.

Diabetes educator: I support what he said. We can also arrange for a group on WhatsApp which must be under the supervision of a dietitian, a diabetes educator or a nurse, as well as supervisors so that if any patient made a rumor while chatting with each other, the doctor or the educator can interfere and correct.

Diabetes educator: I give him options. We recommend a diet for him that does not deprive him, for example, reducing the carbohydrates, in this case, he will not be deprived, but we shouldn't, as possible as we can, reduce the things that he likes and we should diversify the food so as not to get tired of it.

Nurse 1: I am not a nutritionist?

Nutritionist: When the patient first diagnoses, we should talk about these things, to explain to him that his diagnose doesn't mean to deprive him from many things, but at least now we need to calculate quantities, such as dates, especially these things are repeated every day, so we will not deprive you from it, but we should let you know the number you can have.

When we talk about rice, the things that exist among the people, which are widespread in society and which we consume a lot as meals, we make it clear to the patient that we do not mean to deprive him, but we will depend on two things: the quantity and the method of preparation. Even these things are up to the patient himself, many patients come here while being convinced that he will be deprived from everything he likes.

It is our duty to give him something that will be useful and not make the patient feel like forsaken in order to participate on any occasion, trying to distance from feasts. This is very important.

The interviewer: Any addition? The developed countries of the world, such as America, Britain, Australia and Canada, have a fixed educational program, which is how the patient conducts self- management, i.e. self-care for the disease itself rather than needing a nutritionist, or the diabetes educator or nurse, only goes to the doctor. So what do you know about these programs?

Nutritionist: I, personally, know nothing about them.

Nutritionist: I don't know.

Nutritionist: I don't know.

Nurse2: I have no idea.

Doctor: As I said before, I do not know about the program in Britain and America. Of course an initiative like a program would be the right thing, but it was never be applied here, so far in Qassim in particular has not been applied, even what was applied depended on other things.

Nurse 1: Certainly.

Nurse 1: Do you mean a website, for instance?

Nurse1: Ok, if I talked about the things you mentioned, it is ok to be a plan in which we put every title and talk about it in details.

Nurse2: This system should be applied at once.

Nurse2: I mean to depend on his own self as reading of these things.

Nurse 2: Yes, it is possible. Eventually, I feel that some patients do not need to come here.

Doctor: As I said, the program was not applied in full; we tried to apply some aspects of it but we were faced by a problem, which is the support. For example, if I told the patient to check and measure the diabetes level every day, like those with Insulin Bump, I need them to measure the diabetes level 6 times or more a day, he would ask me to give him the segments for diabetes analysis or the bandages or anything else. This is the support which caused a problem for us by not doing it, so I think if the program was applied, it would be good and useful for the patient, and however, the application will be difficult.

Nutritionist: Almost, we try to apply these goals by each person personally, but the results will not be great as I think. It would be successful for some patients, even in a satisfactory percentage. I think that we suffer from the awareness; we still can't make the patient do these things on his own and try to control it by himself.

As a specialized centre or reference centre for the whole area, the patient is supposed to come in short periods, but in the absence of discipline, he takes two or three appointments and walks away. It is assumed that he is disciplined and begins to return again to his nearest place, whether hospital or health centre, but the reality here is the opposite; the patients may need years to follow up here, the problem is not in the shortcomings of the treated team, the problem in the patient's awareness.

Doctor: I think that if there is an effort to sort out the patients, it means that a large segment can benefit from this program, since they are educated, because they know the disease and have awareness, so if there is a sort out such as: questionnaire for the level of education, i.e. is he willing to participate in this programme or not, then we review the feedback.

Doctor: I expect it will be fine for those educated people and welling to participate and follow this instruction.

Diabetes educator: I have a comment on the doctor's talk about the support matter, there are many patients suffer from the cost; the ministry did not provide everything till now, so when I ask the patient to undergo an analysis he says: it costs too much and I can't afford it. Moreover, most of them do not have Diabetes Analysis Device, they can't afford buying it, hence, it will affect as well.

Diabetes educator: Yes, sure.

Diabetes educator: Yes, you are right, but there are some patients who can't follow the program as it is not suitable for them.

Diabetes educator: I think that some patients prefer the easy things, they do not like sitting with other persons to educate them; they just want to serve themselves.

Diabetes educator: It will be effective, Allah willing.

Nurse 1: No.

Nutritionist: I think that this program depends on awareness as a beginning, and of course the educational level which comes after the awareness, because there are many educated persons but they do not have awareness at a satisfactory level that you need to give him the decision in his hands and follow him each a given period. I think there is a large proportion that has the biggest problem in diabetics in the region here at least, which is the awareness.

Doctor: The most important thing here is not to be like the other programs; I mean that the Ministry of Health may adopt many programs such as: Geriatric patient, but the problem lies in the application mechanism which eventually become overload Paper works. The most important thing is efficiency and the mechanism of application, when you say an idea, I tell you it is a very wonderful idea, but how to apply it, this is the problem.

Doctor: You mean a life style program not a soft copy?

Doctor: The first thing for me is to make the patient organize his time, when he gets up, eat and sleep, in order to make the medications related to it. After that, the activity in which he will make an effort so that I may reduce the Insulin. This is the first thing, sleeping, waking up, eating, and exercising should be organized.

I mean that the patient tells me about his life style so that I take the information and we agree on the best thing for him, and then we start whatever plan.

Doctor: The first thing, for a diabetic patient, to fix three main meals; if the period between them exceeded four hours, there should be snacks. Some of them think that I increase their meals, no, it should be healthy meals such as: salad and the alike.

Then I set the Insulin dosage, especially the Lantus, at a specific time; this will help us afterwards. I advice him to undergo an analysis while being fasting, based on this, if the result was under 130, it means that the diabetes is under control. After that I ask him to make the analysis two hours after having breakfast and make my judgment, this is in terms of treatment, but the living style requires at least half an hour a day of walking.

There should be no rest more than two hours a day without doing activities.

Nurse 2: It is a start, how long the patient is diabetic? Type I or II, what treatments does it take? Committed to treatment or not? For analysis, we see whether he practice exercises or not? Committed to diet or not? Accordingly we can make an adjustment if needed.

Diabetes educator: If we talked about sport, what type of sport does he prefer, involving a swimming, walking or running? We determine for the non-obese diabetic, 30 minutes per day divided by 10 minutes if he could do it, and 4 to 5 days a week, the most important thing is not to stop doing exercise for two consecutive days, but if he is an obese diabetic, we determine 60 to 90 minutes a day from 6 to 7 days a week as exercises.

Diabetes educator: For meals, we will try not to make them three main meals as the doctor said, if he wishes, he may have snacks. I mean to we determine, as possible as we can, the amount of carbohydrates so that the quantities are reasonable.

Nurse 2: I can't tell.

Nurse 2: No, I have not.

Nurse2: Yes, but it is different if the patient undertakes a chest medications, I develop my own plan because it is out specialization.

Nurse2: I can make him keen to take the treatment on time, and be convinced of the words of the doctor and convinced of the diet he walks on, and to be keen to review us every day, not every six months or 4 months, and make him promise me to be keen on taking the medicine.

Nutritionist: Of course the plan always depends on the type of treatment, whether it is Tablets or Insulin Dosages, the number of meals will be different. If he takes Tablets, the meals may be three main meals and two snacks, but if he is taking Insulin, the meals will be three main meals and three snacks; it is very important that the patient knows the level of diabetes.

He must be analyzed before practicing any sport, of course colleagues talked about the quality of sport and its sustainability, but there is also an important sign of measuring diabetes before doing exercises, and what proportions through which the patient can do exercises, because there are proportions have serious risk of sugar reduction. Moreover, the patient should know how to deal in the case of exposure to low blood sugar, and what is the base here? What meals should have in order to raise the sugar level, and when it would necessary to go to hospital if there are specific readings? Can he control the level at home? Anything like that, which is important, as well as setting basics for this in order to make the management.

Doctor: I agree.

Doctor: There is also the issue of exploiting the means of social networks in education, for example the Ministry of Health wants professional people to convey the right image, and to make the video clips which are supposed to be viewed and spread within the community, which shall be under the umbrella of the ministry.

Nutritionist: As the doctor said, to try to spread the awareness through the mass media and through the creation of medical teams and a suitable medical team, provided with a full range of specialties involved in the treatment of diabetics in primary care centres. The second thing: of course, the main problem we have in the Kingdom of Saudi Arabia is the food style or, in general, the life style, which is not to practice exercises enough, and also to rely on the qualities of high-calorie foods, high amounts of fat and also high amounts of sugar. I think that anything needs a government program to change it, needs to have a huge program to try to change the lifestyle of the community.
