# Supplementary material for: Cultural adaptation of self-management of type 2 diabetes in Saudi Arabia (qualitative study)
Source: PLoS One. 2020 Jul 28;15(7):e0232904. doi: 10.1371/journal.pone.0232904 (PMC7386581; doi:10.1371/journal.pone.0232904)
Supplement: S2 File — (DOCX) [file pone.0232904.s002.docx]

Focus Group 2

Doctor: For us, every day we see patients, not in a specific time but every day. There are many clinics, so every day you find patients.

Doctor: Well, for the same patient, I may set an appointment according the condition of the patient, some of them come on a three months basis and others come every six months, also there are some patients who come only for consultation at any time. There is a room call the Rivera looks like the analysis designed for consultations.

Nutritionist: According to our job, we come after the doctors directly. When the patient comes out from the doctor's clinic, he directly comes to us at the nutrition clinic without any appointments. We have formal clinics on Sunday, Monday, Tuesday and Wednesday such as nutrition which is a formal clinic for a half of the day and receive the patients transferred from the centre for the rest of the day. So we see the patient after being seen by the doctor.

Nutritionist: Yes, some patients come to us after being transferred by the neighbourhood clinic, which are the formal clinics, as I told you before on Sunday, Monday, Tuesday and Wednesday for the general nutrition clinic, whether Obese or Underweight, whatever the health problem other than the diabetes, but the rest of the day is being designated for the diabetics at the centre here.

Diabetes educator: For education, we are all here come after the doctors but under the same nutrition system. However, some patients come here for consultations, so I determine my own appointments on which I can set an appointment for the patient if I felt that his condition needs follow up, so that he can follow here in terms of dosages, ……..

Diabetes educator: Sure, most of them do, and others communicate with us in order to give him the telephone number of the clinic or my own number, or communicate via text flyers so that I can know about his condition, especially if he is a fresh diabetic or in taking medicine, if there is injection or anything new for him.

Nurse: We see patients every day, because it is the system of the clinics and also there is booking, so every day we have patients.

Nurse: Yes, they are.

Doctor: It depends on booking. Sometimes the clinics are overcrowded, i.e. 20 / 25, so we try to give them the basic needs. The nutrition clinic also helps us, so if I want to talk about a treatment, I transfer the patient to the nutrition clinic, as well as the education, for example, if I wrote injection and new meals, I send the patient to the education specialists who help in making the patients understand the part which I couldn't make them understand it at the clinic. Moreover, they may help me outside the clinic when I transfer the patient after the clinic to the nutrition and education. I try to talk only about the meals in a short time as far as I can in order to convey the flyer to the patient in a short time regarding the treatments and the alike.

Doctor: Of course if the Booking is not too much and the clinic is not crowded. Instead of checking 20 patients, we'd better see 15 or 13 patients only so that we have enough time to sit with the patient and hear about his needs and concerns if he needed to enquire about anything. By this way, he would take his right time if the Booking was not too much. This is the solution from my own point of view.

Nutritionist: For us, as a nutrition clinic, we talk more with the patient, because as you know that the nutrition is the base of everything. When there is a diabetic taking medications and another diabetic does not take medications, is they are not on an exact diet, the diabetic who takes medications will not benefit from the medication and the one who does not take medications will be worse and will be enforced to take the medications.

In order to convince the first patient the correct eating habits and take the dietary habits, which the patient originally follows, and begin to improve them and tell him what is right and what is wrong, and then calculate the calories and put him on an exact diet, all this will take much time. The same thing in normal days when the Booking is more than 20, I feel stress, but if the Booking is less than this, I would have enough time with the patient, especially regarding the nutrition which requires convincing the patient, if the patient is not satisfied, he will not follow the diet.

Doctor: It is the same thing, all of which relates to the organizing process; if the number of patient is few and there are more clinics, it would be better than being stressed in a few clinics with too many patients. This is considered as good solution, I mean to distribute the patients on more clinics, instead of 4 clinics, it would be better if we have 6 and 8 clinics.

Diabetes educator: For us as educators, the time depends on the patient himself, meaning:

According to my study American diploma studying by the way, Institute related to Harvard University, what they said?

I should ask the patient about his own time, to take permission from him, do you have enough time to explain so and so for you? So, I should ask about his time, if it was not enough, I only give him the basics. Also, the purpose from the visit; assume that he came today only to learn about Injection, I should only explain the injection and focus on it because his time is not enough, and then I give him an appointment if I felt that the patient does not need awareness, but if he needs awareness about other things, I gives him an appointment to come to me.

Diabetes educator: When there is too much Booking, some patients feel stressed and the time is not enough. Sometimes, if the time is not enough, I feel that I did not give the patients all the information he needs, and gives him an appointment for the next day; hence the booking increases to 20 or 27, so I give him an extra appointment in order to complete the things he needs.

Nurse: In my opinion, the booking should be few as they said, because the booking reaches 25 or 30 cases in some days, that is why the patient does not take his right time at the clinic because every patient has a definite time, and if the booking is too much, it will be difficult for the patient to spend long time at the clinic.

Nurse: To reduce the booking, increase the clinics and increase the staff so that every patient takes his full time.

Doctor: For patients, the communication is on time, the time they come here on which their appointments are determined. However, we have another idea named the transfer room, it looks like the consultations; for example, if I determined an appointment for the patient after 6 months, the patient is not forced to wait for these six months if he has a problem regarding the treatment. If he comes to tell me that he does not know how to take injection, or that if he said that he had the medicine which I wrote for him but he had side effects, he does not have to wait for the six months, and may come at any time to an open room or emergency room.

Doctor: No, it is not. It is in the morning from 8 to 12 pm.

Doctor: Do you mean what time does the patient come?

Doctor: No, he does not. It depends on completing what he need. Every patient is different from other patients; some of them may take 15 minutes, others may take 5, and there are some patients, for example, tells me that he had a medicine which caused a cough, so I change this medicine and give him another, which may take two or three minutes. Other patient needs more explaining if there is a problem; for example, if he is a diabetic and needs to take his tooth but the dentist told him to check his diabetes first, so he comes to me without any appointment in order to give him medications and consequently he takes more time than others.

In particular, there are female patients who are pregnant, and it is important to control the diabetes throughout the pregnancy; those patients can't wait for six months, she must come to this open room, to the transfers at any time once she knows that she is pregnant. We ask them to come once they know that they are pregnant in order to check them, control the diabetes and undertake the necessary analysis and treatments from the beginning, so it is different, there is nothing as it is.

Nutritionist: For us, as a nutrition clinic, when the patient come to us transferred by the doctor, we start diagnosing the case, and set the appointments based on the condition, i.e. we are not obliged to be linked to the next visit which is after 6 or 7 months. Suppose that the patient transferred by the doctor is DN2 and suffers from obesity, fat problems and stress problems and so on, I should follow up the patient in my clinic at the official days, which are Sundays, Mondays, Tuesdays and Wednesdays.

Sometimes, Under Weight patients come every two weeks. Therefore, according to the condition of the patient, I give the appointments related to the nutrition away from those of the doctor's. It is only at the first time which we should be related to the doctor's appointments; afterwards, we set the appointment according to the needs of the patients from our point of view.

Nutritionist: We like to communicate with them just like the nutrition clinic and with the appointments set by the doctors, but there are exceptions at some times when it is necessary to communicate with the patients.

Nutritionist: For example, there is a patient with Dialysis and Diabetic, so I had to communicate with her via WhatsApp in order to observe her condition with her. She used to brief me about the rising level of diabetes till I could control it to reach only 300, so I have to follow up these critical cases.

Nurse: For us, as nursing, we could help the patient when he comes to us by checking out his accumulative or fasting diabetes level according of course to an order by the doctor; this is what we can provide for the patient.

Doctor: Of course, the most important thing is the analysis, the main thing that the analysis of the patient is the one that directs the patient; for example whether the patient controls its diabetes condition, whether the medicine have a good result or not, whether the patient takes his medications regularly or not and whether he takes the right medications or not. Therefore, the most important thing is the analysis for us to make us able to assess the condition of the patient whether he responds to the medications or not and whether these appointments are useful or not for him.

Doctor: What do you mean by "to be checked"? Do you mean to know the result?

Doctor: No, he comes to know whether the diabetes is controlled or not.

Doctor: Certainly, because the analysis is the most important thing to him. When the patient comes to the clinic but did not do the analysis, we tell him to do the analysis in order to benefit from the appointment. So, the analysis is important in order to take the medications and know whether the diabetes is controlled or not.

Nutritionist: As you know, the Obesity may represent 90% of the causes of DN2. If we measured it in an exact way, you will find that Obesity is the main cause, so through the dist, I can follow up the patient and let him know that he will get better after six month after doing the accumulative analysis by the doctor. I told you previously that I make him follow a diet; I then follow the patient through losing weight or through follow a diet or changing habits, hence, through the follow ups, I know if he will respond in the analysis eventually. Moreover, he has a device at home, so I am very keen to make the patient follow an exact method in meals and tell him to do the analysis two hours after the meal and have a look at the result.

At home, the patient finds out that his diabetes is controlled when he eat properly, so he is reassured. We make the patient understand what the HbA1c is, and to know what the accumulative diabetes is, as well as how the diabetes affects him, this is what make the patient, as the doctor said, keen on doing the analysis before taking the medications. In the past periods, the patients used to come here only to take the medications and go home.

However, with the education of the doctor, they began to understand; also we started to make him understand what does the high level or low level of diabetes mean? What would happen if the patient committed to what we say? How far do you avoid the complications in your body when you attend to us? The patients, Mashallah, come here insisting to do the analysis, as the doctor said, then comes the medicine.

Moreover, the aim is to tell the patient: our next goal when you see the doctor is to reduce the medications, when the patient sees the doctor and the doctor tells him that his condition is good and started to reduce the medications, the patient knows that he is doing it right, but on the contrary, the doctor may intensify the medications if the patient condition is not so good. We decide this through his follow ups with us every month.

Nutritionist: Of course there are some patients, especially the young people; keen on following the instructions, perhaps it is because the education and thinking are different from older people's, while the older people are some kind difficult to be convinced because you want to change their life style to which they are accustomed, so they may take some time to be persuaded.

Those patients, who are 30, 40 till 50 years, are very responding, their analysis are getting better and the doctors feel satisfied with them as they follow the directions.

Diabetes educator: We have the same system. We provide the support for the doctor and the nutrition. The doctors often prescribe fructose for the obese and Type 2 patients, so we follow with them so that we notice the weight. Also, we make explanation for A1c and make them understand what is the purpose of it, and how much the target that suits the patient? It varies from one patient to another.

Diabetes educator: I feel that they are getting better; I mean we make some progress.

Diabetes educator: It is education or the age. Sometimes, the elder people understand more than the younger ones, and other times the younger persons may understand all what we say and follow it, so it is according to the patient himself.

Doctor: I'm not convinced of the Social Media, there many things that are not true. We face a problem when the patient comes and says: I read on the Internet that this medicine cause so and so or someone told me so and so, it is not everything. Each patient is different from the other, they may sit together and hear that a given patient takes a given medicine, so they come to the clinic and ask me why I did not write such medicine for them.

Of course the medicine that benefits a patient may does not benefit the other. However, they are convinced that if such medicine benefits some patient, why don't they take the same medicine? as they hear from other people, but I refuse.

On the other hand, some of them tell me: I logged onto the Internet and found that this medicine has side effects and other things. Any medicine has side effects written on the pamphlet, but they happen rarely. I always tell them to attend only to what the doctor says, not everything on the Internet is right, not everything written on the Internet is correct.

Their problem is that they surf the Internet and understand that the words of the Internet is better than Doctor's.

Doctor: Other than the doctors, there are for example the awareness programs. We go many places and Malls; there should be something like Diabetes National Day. There are education sessions with doctors, what if the patients attend those sessions and cared about it. Moreover, what if we have flyer and flyer, when they ask us, we answer; I mean to have a reliable source, not any source, it should be a reliable one, such as the awareness programs. This is the most important thing from my own point of view.

Nutritionist: As for me, as nutrition, if you come and see the nutrition clinic, you will find many forms of awareness which I use in many things.

The first thing, for example: The food palm and we have the plastic samples and the size of dishes, the size of spoons and the size of the cups which the patient would use; of course it is not random. We say, for example: when the patient enters the clinic, calories are to be calculated for him, and through these calories, I determine the amount of food that he would have in each meal.

In order to determine the amount of food that he would have, I must let him see it himself; i.e. when he came to the clinic, the plastic samples here would help me in such matter. Also, there is something that I like to do with the patient, which is to record video and audio video clips for the patients; when a patient responds to the nutrition with the medications he takes, whether it is diabetes or pre-diabetes, he can notice how this patient achieved a good result with the diet. I record the video of the patient video and audio and show it to other patients who come later, of course this through the approval of the patient, so I make this video today, for example, so as to show it if there is anyone not convinced that nutrition, education and walking have effects with medications of the diabetes, because the majority of the patients think that medications only is enough. Other people may say: once I take the medications, I may eat whatever I want and live my life as I like; the result of course is that the diabetes level is high and everything was to no avail.

In order to convince people with these things as I said to you, I have the visual aids in the clinic, and the videos that I record in the clinic which are audio and video for the same people here, and so I think that it is to be considered as persuasion of the patient.

Nutritionist: Thanks be to Allah, the centre here provides us with almost everything we need, even when we carry out awareness campaigns, the centre and the nutrition division within the hospital provide help for us, anything we need such as flyer or flyer. Also, we recommend diets for the patients, as I told you, and prepare many things inside the clinic, all of which is provided to us by the centre, so we need nothing from outside.

Diabetes educator: We have many educational tools, and we have, for example, the "Expired of some medicine" in order to explain for the patient the Insulin bump, so that I can explain for the patient and make him try it in front of me before injecting himself, especially the newly diagnosed patients, as well as having a schedule in order to make sure that he undertakes self-control at home. I also teach him how to use the device, whether it is Glucometer, Liber or Insulin bump; we teach them the same things we do which they should do. Moreover, we have flyer, flyer and booklets.

Diabetes educator: Certainly, as a new plan that has its pros and cons because I read about this, and there are some people who tried it from other groups belong to our study, who said: if the same patients heard their ideas, for example about the injection, and imitate one another in negative things, for example: a patient says: I did not take my injection and nothing happened, so other patients would imitate her and take negative and positive things from one another, but it is truly everything.

Nurse: I answered on the same group and its efficacy, because they used to exchange opinions with doctors, educator or nutrition specialist. They talk to each other, every one of them talks about the negative things he have as well as the positive things, but they used to imitate each other in negative things.

Suppose that the patient has no such thing, she may skip the medication.

Nurse: Certainly, it happens through the open discussion; when a patent says such thing, the educator asks her why does this? Because the so and so said this in the group, so they advise her.

Diabetes educator: We already created groups such as Insulin bump; there is a new nutrition name carbohydrates for diabetics, especially those type 1 and type 2, in which there are the doctor and the educator along with the patients in order that if any patient asked any question regarding nutrition, I join the group and answer; if there is a question related to the medications, the doctor would reply.

I think that it is a good thing; if any patient asked a question, the information goes for everyone, but we apply this only for the Insulin bump which is for the people of the group; the rest of persons are through the WhatsApp.

Diabetes educator: Yes, it is very effective. You may find that some patients call us, communicate with us via WhatsApp, ask their questions and we answer at once. I think that WhatsApp is easy to use, why is that? Because it is always available, and the patient may ask any question at any time, so when I see the question, I reply; it does not require appointments or requiring the patient to come or go, so I think that it is nice and useful.

Diabetes educator: Yes, it is good according to the comments that I hear during our study today.

Nurse: I think that the WhatsApp is a good idea, and already applied. The patient can follow up with the doctor through it, even though the Holy Month of Ramadan, when the diabetes gets low due to fasting for long period, the patient can follow up with the doctor to adjust the dose through the WhatsApp without asking the patient to come to the centre, so I think that it is a good, quick and practical means.

Diabetes educator: Yes, there are the screens, for example, to be in the waiting areas for males or females as boards in which we can show the symptoms.

Diabetes educator: In the waiting areas mostly.

Diabetes educator: Why not?

Diabetes educator: Like Malls or public places such as the parks.

Doctor: They may be a scientific meeting in any hotel in which we make a group for the patients to communicate with each other, such as the lecture, and the doctor is there along with the educator and the nutrition specialist; if any patient asked a question, any problem he faces in the treatment, everyone else would hear the answer and benefit from other patients and the answers in which everything would be like an open meeting or scientific meetings, for example, to be determined once every month.

Nutritionist: There is also an idea which we applied in the centre, the schools and universities. We always have meetings from the schools and universities in which we accompany, as a group, the doctor, the educator and the nutrition specialist; we attend these meetings and give lectures for nearly one or two hours, and the school make all students available for us to give them complete awareness regarding the diabetes and its symptoms. In order to start the awareness correctly, you should start from schools, because every a single home has a student or two, or may be more, in schools; so when there is some in the family with diabetes, the student will take the information from us and transfer them to his home. We try to make these meetings attractive and not boring in order to attract the students who want to understand. Also, we take flyer with us and distribute them on the students; and we determine a given hour after the lecture for those with diabetes so that they can come to us and ask whatever questions they have, so I think it is something good, as awareness, and I liked the idea.

Diabetes educator: Of course, if you allow me, there is something related to the neighbourhood dispensaries as they deal with patients more than anyone else, more than us as specialized persons; the patient always go to the neighbourhood dispensaries first, but when he tells the doctor that he has diabetes, the doctor transfers him her to the diabetes doctor. They should carry out awareness for those doctors, I mean of the neighbourhood dispensaries, along with meetings, education and awareness more about diabetes so that they can benefit the patients. You know that the Booking is crowded and always very busy, why don't we start with those doctors so as to provide awareness for the patients regarding the diabetes and its problems, so when the patient comes here to the diabetes centre, he would have enough awareness and take the rest of awareness from the educator here at the centre, this is an important point as well.

Diabetes educator: Of course we educate through complete exercise, every patient depends of himself. For example, I can't make a patient with heart problem do exercises because it will not be fit for him, I may make him walk or go swimming or something like this. Also, there is the wish of the patient; I am not talking about the time of the patient, I mean to ask his permission and look first at his wishes, we try to help him as per his condition and wishes. Some of them can do a particular sport, but don’t like to do it, so we replace it with another sport.

Nutritionist: To make him like it through showing photos or flyer, or we tell him about the benefits and the risks in order to make him like the sport; some of them respond while others do not. If some patient suffered from problems with nerves and diabetes, I tell him that swimming is the best sport for him; if he said that he does not know how to swim, I tell him to go to the club and make a group of friends and go together; it is some kind effective with some patients and they do respond to such thing.

Doctor: As diabetes centres, I suggest that there should sports hall which should be under supervision and according to ages of the patients, in order to guide the patients.

Nurse: We may do so in the dispensaries of the neighbourhoods or in the clubs at schools, such as those of type 1, the younger patients, but the elder ones should be in the dispensaries of the neighbourhoods. This what I expect because most of them can't do so, or has no place to walk on, or even has no one to accompany him, so I suggest to be at the dispensaries of the neighbourhoods or …..

Doctor: Of course sports is very important for the diabetics, it is a basic thing; sports and diet are more important than the treatment, they are ABC for treating the diabetes, so it is very important for them; they can hear experiences from each other, as she said, I, myself, when I watch a video for someone saying that he followed a diet and practiced a sport which made him lose his weight or got his diabetes lower, and now he takes medications no more after following the diet and the sport, this would be a means, for me as a diabetic, to act like him.

The centre here should have another building containing a hall with a Gym provided with a track in order that the patient can walk on; but there is nothing of this till now. The diabetes centers itself can have some kind of clinic, such as the Physiotherapy and the alike, those patients do not book appointments to go there; why don't the diabetics have the same? Why don't the diabetes centers have something like the sports club with a supervisor or someone else specialized like the nutrition specialist? In order to be with the patient or beside the nutrition clinic so as the patient, after taking the proper diet from a responsible person there, can check whether his weight got lower or not.

Nutritionist: For me, as a nutrition specialist, in order to encourage the people to walk, I tell them to walk at any time; for example, if the patient said that he is busy, I will tell him to walk at night, at dawn or at any other suitable time.

Moreover, in order to encourage him to walk, I ask him to walk for only five minutes as a start; when you tell the patient to walk for only five minutes, he will say that it is simple and easy, but it is also according the condition and the age of the patient, because elder patients can't walk as much as the younger ones; then I ask them to increase the time of walking, I ask them to do so gradually.

Also, there are patients whose fats are not burnt unless they walk, so I tell them about this in order to be convinced. In addition, there are so many patients want to lose their weight, I tell them that they should do three things: a diet, drinking enough water and practice walking, with these things they can burn fats and benefit from it.

Diabetes educator: The same thing, it should be done, but as I told you about the Obese who have problems with the nerves.

Doctor: We should carry out some activities on the National Day of the Diabetes, in malls or schools.

Doctor: Yes.

Doctor: I recently read on Twitter that there was a walkway in the housing area, in which there was groups walking.

Doctor: Yes, it is effective with the teenager and the adults; I expect that they would respond to such thing.

Doctor: Even the females, we ask them to walk in parks, in the walkway at the gulf or in the walkway of the housing area.

Doctor: It would be in parks, gardens or walkways, to make a group and walk together.

Doctor: Yes, I do, but in public places.

Nutritionist: If a price made an invitation, at the level of Al Qassim area, for example, for the diabetics that he assigned a definite place for male diabetics and another place for females, so anyone wants to go, would go. This is how you can encourage the people to walk more than us, because we have limited resources as a centre, and if we thought about it, we would find no support. It is the same problem.

Nutritionist: If there is support, it would fine, because you will provide the place and the sports trainers and hence benefiting the patients.

Nurse: He is not talking about walking only, he talks about sports, all types of sports.

Diabetes educator: I think that the female patients here in Al Qassim area will not come, let us be real. We ……..

Diabetes educator: Because of the culture, no one would go out to walk while wearing the home dress. So, I think that if such thing was made in sports clubs for example, which contains everything such as walkways, tracks or such things that exist in the sports club in a closed place, it would be better for the female patients; I am talking about the female patients in terms of culture.

But frankly, if I told a female patient to go out and walk, none would come.

Doctor: They will not come, it relates to the culture.

Doctor: I suggest providing a closed building, such as the sports club, for the female patients, in which they can do every sport in such club, such as swimming, walking or other things, provided to be in a closed place, but if the marathon was made in an open area, I think they won't come, even if it was the Health Day.

We may ask them to do sports and have healthy meal, by doing so, we make them benefit from these two combined things; I mean to know what the healthy food is and to practice a sport.

Nutritionist: I expect the contrary, as you know, we live here in a closed society in which women do not like to go out for walking, there would be men, an as the doctor said, the female would go out wearing the home dress or the veil, she will be upset, but if we provide a closed place for her and told her that this place is assigned for the female diabetics, she would go. As you know, the Kingdome of Saudi Arabia comes at the top in diabetes on the world, so it is supposed to have more awareness, to assign places for the female diabetics and other place for the males in order for practicing sports and spreading the awareness and nutrition to them all.

Doctor: Of course, the majority of the diabetics are females due to wrong traditions, there is no movement, and they sit at home most of the time. They should go even to relief themselves and to teach their children how to do sports. For example, if the young children learned how to swim, it would benefit them and decrease the obesity and diabetes. So, this is very important thing, it would be a good idea as I think.

Nurse: I agree with connecting the clubs with the health centres, because patients need to do sports, but not to tire themselves in order for their blood not to mix with Hypoglycaemia, there should awareness and supervision by the members of the club.

Diabetes educator: I suggest that the diabetic should apply for subscription in a club in order to be encouraged more from my own point of view.

Diabetes educator: Yes, to be provided by the centre, I suggest.

Doctor: I just want to explain something, it is normal here to see females walk in parks and gardens; on the contrary, there are so many female walk on Ramadan and on the Feasts (Eid), I was surprised by their numbers because all the walkways were full of them, I mean that it became something normal here.

Doctor: No, it is only aesthetic matter.

Diabetes educator: I want to explain that the veil is not an obstacle; the female can walk with it even in a public place.

Diabetes educator: Yes, that is right.

Nutritionist: But some patients have no potentiality to go to the health club, such as subscription or the financial ability to go there, in particular, most patients have no ability or potentiality, so I suggest that this month, for example, we choose a specific number of patients.

Doctor: We could make it as a competition, for example, we tell the patients that the one that can lower the Haemoglobin or the accumulative diabetes to 8 or 7 for example, he would get a month subscription for free in a club, so that all patients would compete in lowering their diabetes level because there is an incentive or a reward for them.

Doctor: Or may as a discount.

Nurse: Good idea, so if the patient went to the club for a month, for example, he would get used to walking and will find himself relaxed and better than before, so he will start to go to parks to walk. So, I think that we should start, as the doctor said, to provide a reward or a bonus for the patient so that he continues doing the sports.

Diabetes educator: Yes, of course, all what you said right now are the basic things in which we are talking theoretically and practically, they are the basics that would enable us control the diabetes or prevent it from hitting the people.

If every patient, since he was young, was educated about the diabetes, how to walk, how to do sports and how to eat healthy food, he will not suffer from the diabetes, and diabetes will not be spread so widely in the Kingdom of Saudi Arabia here.

The way you eat and wrong habits in general life may raise diabetes, all this points should be covered, if they were applied, the people would know how to protect themselves and how to treat themselves in case of having diabetes.

Doctor: Of course they are the basic things to be developed in order to provide motives for the patients, pre-diabetes, diabetics and also the non-diabetics in order to improve the life style.

Nutritionist: Sure.

Nurse: Of course they are the basic things that we should tell the patients about, and certainly they are effective.

Doctor: I want to add that as we talk about the diabetes, we did not mention the diabetic foot, which is very important, I mean diabetes complications. Diabetes affects the nerves, kidneys and eyesight more, so we could increase the awareness for the patients in terms of eyesight or the diabetic foot, how to take care of the diabetic foot, how can the diabetic protect himself? How to preserve care in order not to be affected by the diabetes? How to protect his feet and eyes? As well as kidneys, that should be cared about through doing analysis, and with the doctor.

Nurse: I think that the diabetic foot is very important because the patient can control it.

Nutritionist: I have a suggestion for people or for dependants, which is not to wait till I have diabetes so that I can go and make analysis and checks, we should make it as a culture for all the family, for example once every 3 or 4 months; perhaps they may find out things which may cause harm when discovered later. Hence, there should be medical check-ups for all people, whether children or old people, constantly, because it would be good thing.

Diabetes educator: I agree with these goals, diabetic foot is essential, and healthy people as well, they should undertake a regular check-up as the nutrition specialist said.

Doctor: He should first understand about the program and its aims; I, as a doctor, should see this program first in order to make the patient understand and in order to tell him about the benefits of the program in order to be convinced and join it.

Doctor: Yes, for example, we can make a group from the patients and make it as a scientific meeting or a lecture so that the patients may sit together and understand the program and that they would benefit from it if it was applied.

Doctor: In my opinion, if we gather some educated diabetics, as you may find a doctor with diabetes, or a teacher with diabetes, so we could gather these people together and let them talk to people in order to improve their behaviours and let people know that these persons are just ordinary people, so they can persuade the other patients more than us, so if we made a group of patients with the same illness, while having good level of education, we can convince people that it would be a good thing.

Diabetes educator: Yes, of course. Under the supervision of doctors of course, and the doctors would choose them and the points about which they will talk.

Diabetes educator: We applied this thing already at the centre here with Insulin Bump when we attend the Insulin Bump course; we brought an employee with us with Insulin Bump, and shared the same thing and his experiences and could he maintained his pump, and it was effective and very useful.

Diabetes educator: We could make videos in an easy way in order to be understood and put them on the YouTube and send them to the patients or on the WhatsApp in order to benefit from it and how to make use of it.

Nurse: All confectioneries, malls and supermarkets must have awareness.

Nurse: Yes, to provide explanation……

Nurse: If there are clubs that contain enclosed basins or closed hall in order to make the person stay at the club.

Nurse: There are no swimming pools.

Diabetes educator: I want to add that if the patient was prevented from doing something, he would be keen of doing it, so the first thing we should do is to make him feel assured about his illness, not to make him feel that it is the end of the world. We should make things easy and simple for him and make him understand that it is just a lifestyle but it changes, treat him as a friend or a brother not as an illness.
