# Supplementary material for: Cultural adaptation of self-management of type 2 diabetes in Saudi Arabia (qualitative study)
Source: PLoS One. 2020 Jul 28;15(7):e0232904. doi: 10.1371/journal.pone.0232904 (PMC7386581; doi:10.1371/journal.pone.0232904)
Supplement: S3 File — (DOCX) [file pone.0232904.s003.docx]

Guest: Walikoum Al Salam.

Guest: Alhamdulillah, I’m on the diet they told me about. They say I should have one grain of fruit per day. What’s the second question?

Guest: Yeah. I always eat leafy vegetables as they don’t raise the diabetes.

Guest: Yes, I eat vegetables more than fruits.

Guest: I eat them daily in the morning, noon and evening.

Guest: I rarely eat junk food.

Guest: I rarely eat it because of the diabetes. As it consists of fats and carbohydrates as you know.

Guest: Yes. I avoided eating it after having diabetes.

Guest: Yes. I walk daily.

Guest: I feel that walking reduced diabetes.

Guest: I walk every day.

Guest: Not less than 20 minutes every day.

Guest: No, I liked walking before but I was not keen on it. Now I am walking when diabetes rises and I feel comfortable after that.

Guest: The first source is the dispensary. They are the ones who teach us and tell us whatever we want to learn. I know people at home who know about the disease and they teach me.

Guest: I take care of my health.

Guest: I was psychologically tired after being diagnosed with the disease, although my father suffered from it -may his soul rest in peace- But Alhamdulillah I accept it now.

Guest: No, the dispensary gave us Diabetes Device. I measure the diabetes daily in the morning and after every meal.

Guest: If the meal consists of fat, I avoid it and do not eat it.

Guest: it destroys the body.

Guest: Many sweets, too much starches and fats are harmful. This harms the blood sugar level and raises it.

Guest: As I said, I was down for couple of days then I thought about it again and said “Thanks God”. It’s not a serious disease and if one takes care of it, he will deal with the disease.

Guest: for me, they are not difficulties. I ask for tea without sugar and they bring me what I ask for.

Guest: No, I don’t think like that.

Guest: if they bring me tea with sugar, I will leave it or I will tell them to bring me one without sugar if they ask.

Guest: I don’t have concerns Alhamdulillah. Since I had diabetes, I have never got dizzy. Sometimes I have headache and I take aspirin which is not dangerous. I did not have a fainting too, so I have no concerns about the disease.

Guest: Sometimes from the television, the mobile, the dispensary or people who teach me.

Guest: any food with the brown wheat not the white one is the best.

Guest: Vegetables and fruits as I said before, especially salty fruits such as kiwi.

Guest: Thank God I take care of myself and my health. I advise my relatives who have diabetes like me but they eat whatever they want.

Guest: No, I just walk. I do the household, too. I don’t swim, I just walk every day.

Guest: Yes, I want anything related to the diabetes. I want to practice in the afternoon or the evening.

Guest: Yes.

Guest: Sometimes I walk in the pedestrian lane or in my house.

Guest: Yes, always. I never walk indoors.

Guest: I don’t walk in winter because of the weather. In summer, I go out when the sun sets.

Guest: Actually I walk more in summer. I walk for one hour in the walkway.

Guest: There are many walkways that are available for both men and women. So it’s the best for us to walk.

Guest: you know the pain the legs and the back.

Guest: Guiding and teaching us helpful things are good.

Guest: The exercises.

Guest: I want them to direct us to the movements.

Guest: In order to do such movements.

Guest: No, I don’t Alhamdulillah.

Guest: its daily form?

Guest: do you mean the time?

Guest: The evening is better than the afternoon. I want all the food to be healthy.

Guest: Fruits, vegetables and anything that is free of fats. They are useful.

Guest: Yes, I have changed my diet because of diabetes.

Guest: yes. I avoid eating sweets, fats and starches.

Guest: No, the treatments they gave me are good. They made me feel better. The vitamin makes me feel sick so take it every 3 or 4 days. I'm supposed to take it daily.

Guest: No, I take pills. Organizer, its strength is 25.

Guest: twice a day.

Guest: its strength is 500.

Guest: Yes, I take it in the morning and at night. At first, they gave me 3 pills but now I take only 2.

Guest: I want Diabetes clinics to be available daily. Because they said you should reserve before coming as you should have an appointment like the hospitals, and you know that diabetics may need them suddenly.

Guest: Yes, this is.

Guest: Thanks, you too, God willing.
