# Supplementary material for: Cultural adaptation of self-management of type 2 diabetes in Saudi Arabia (qualitative study)
Source: PLoS One. 2020 Jul 28;15(7):e0232904. doi: 10.1371/journal.pone.0232904 (PMC7386581; doi:10.1371/journal.pone.0232904)
Supplement: S4 File — (DOCX) [file pone.0232904.s004.docx]

Guest: hello, may Allah grant you greetings?

Guest: all praise be to Allah. I'm good. I keep eating the fruits and vegetables continuously because the doctor advised me to do that. The doctor advised me to eat the fruits which don't contain more sugar. He advised me not to eat banana and grape. He also advised me to eat little of the other fruits not to complicate the diabetes.

Guest: I swear by god that I don't keep eating them. Openly, I don't keep eating them. Sometimes, I don't eat them. When I want to eat, sometimes I find nothing to eat except the fruits.

Guest: no, no, by god. Telling a lie is non-success. I don't keep eating them daily. Whenever I think of eating them and find something to eat in front of me, I eat it.

Guest: between you and me, I swear by god that I feel pain in stomach and colon, so it is not allowed for me to eat the fast foods. I'm not interested in fast foods to eat them every day. I eat these fast foods rarely.

Guest: no. I eat fast foods since a long time, but they are not my favourite foods. But I didn't decrease eating fast foods since the doctors told me that I had diabetes. I eat whatever I feel appetite for. I eat sweets and other things… I eat everything. By god, I eat.

Guest: I feel pain in my knees, so I think of walking after that. I walk somewhat. The doctor advised me to walk for half an hour in the beginning at least gradually. The doctor told me that next I can add half an hour of walking, and after that I can increase walking.

Guest: I walk for half an hour or an hour every day. I have just started walking. Before that, I didn't use to walk, but I started to walk when the bones doctor advised me to do that.

Guest: no, I swear by god. I started walking after I had diabetes. When I suffered from diabetes, the doctors advised me to walk. I didn't use to walk at all and I didn't try. So, my mistake is that I didn't try to walk.

Guest: the source is King Saud Hospital. The good doctors of the hospital told me everything and taught me the best way to deal with the diabetes. But I didn't deal seriously with that matter. Firstly, I was busy with my children and with their affairs. Secondly, I don't have free time. I feel tired after I come from the faculty and start to do works of the house. After I finish doing works of the house, I don't have time to walk. I pray the evening prayer and sleep, so I don't have time to walk. Now, I ask Allah to help me, the time pass in spite of me and I can't walk or go.

Guest: I swear by god that sometimes I need something called and at times I need something called the psychological factor which can help. I help myself by myself and that means that I don't need someone to help me. This means I know that this is wrong, this is good and this is bad, i can help myself. But sometimes I think suddenly of something, as a type of carelessness, and say that if Allah willing I will be good, but suddenly I suffer from diabetes.

Guest: I swear by god and ask Allah to reward one of my daughters who was graduated from the Nutrition Faculty. She started now to support me and prepare healthy foods for me. She tells me about the permissible and impermissible foods and about the things to use or not to use. She advises me to walk. I swear by god that she shouts to my little daughter to help me to walk. But I say that I will walk. Now, I have no time, and I don't know how the time is lost.

Guest: I swear by god that I know that it is a chronic and dangerous disease, and I ask Allah to save the Muslims from it if Allah wills and doesn't affect even the enemy, as sometimes I feel horrible dizziness and headache not imagined by anyone. I feel fever in my legs and ask Allah to help me. When I told the doctor that I suffered from something, he told me that the diabetes damages and wear the bones away. I advise the people who don't suffer from the diabetes now to take interest in healthy food.

Openly, I was stubborn at first. But now stubbornness doesn't benefit me. Nothing benefits me. I must be enlightened and educated. It is a must to go to the educational centres. Our mistake is that we didn't go to educational centres teaching us about the diabetes and we don't know what it is. When I became old, I went to the educational centre and to the King Saud Hospital to learn about the diabetes. All praise be to Allah that I became improved somewhat. I still eat … telling lie is non-success, I swear by god that it is non-success … this means that it tell lie!!! No I swear by god.

Guest: oh my god. I thank and praise Allah. I was not unable to bear. It is the destiny preordained by Allah. I have suffered from the diabetes since I gave birth to my last son. The doctors told me to take care of my food. I followed a diet for a period after the fortieth. I followed it for a period of time, but I don't know whether I followed it well or not, but I felt dizziness on that dat. My husband, may Allah forgive him, carried me to the hospital and the doctors advised me to give up the diet.

The proportion of glucose is so high. The doctors told me that I must be referred to the specialist doctor. The doctor said that I'm pregnant and suffer from the diabetes. The doctor asked me to follow up. Now, I don't follow up. They said that it is a must to be referred from the emergency to the diabetes clinic. They asked me to stay for two days in the hospital and adjusted the proportion of the glucose and gave me tablets. He gave me tablets and drugs for adjusting the glucose.

He gave me drugs for adjusting the glucose 500. I thank Allah that I continued using it for three or four years and I felt better. I followed the instructions of the specialist doctor, but suddenly the proportion of glucose increased after my husband's death because of the problems of my children and other things. These troubles increased the diabetes. The drugs of adjusting the proportion of glucose are no longer useful. The doctor said that I had to take medicine together with those drugs as these drugs are not enough. The doctor referred me each six months to the hospital. I go to the dispensary continuously.

The doctors in the dispensary said that I had to go to the doctor who gave me the medicine to examine me and find a solution. I went to the King Saud Hospital again. The doctors of that hospital gave me medicine as well. The prescription given to me is two tablets for the diabetes and 2 drugs for adjusting the proportion of glucose. This means that I take 1000. Thus I increased 30 or 40 g. but I swear by god that I don't know the second medicine which I used for three years until that time.

This year, proportion of the glucose increased strongly. This means that 450 are normal in case of sitting. Sometimes I wake up to find the proportion of glucose is high although I ate nothing, and I don't know what happened. I came back to the King Saud Hospital and the doctors said that the only solution is to take the Insulin. You reached to a case that it is a must to take the Insulin in the night and in the day as long as you walk and take tablets normally. This means that you should work during the day going and coming well during the day.

I told them that I don't know how to deal with my case of diabetes; it is high during the night and low during the day …. I don't know!!! The doctor asked me to continue taking insulin till he examine. Till now, I didn't go to the doctor again. As for insulin I take 30 and I take drugs of adjusting the glucose 1000 in the night. As for the day, I take the drugs of adjusting the glucose and the medicine, and this is the full story.

Guest: the proportion of glucose decreased once or twice in my house, so I thanked Allah for that. I don't take part in gatherings. Once, the proportion of glucose decreased during the night and was 35 and once it became 50. My daughter, may Allah reward her, saw that I became sweaty and I said to her that I want a cup of water. She told me that colour of my lips became blue, so I asked her to bring the blood glucose monitor.

I said to her that I want to take the medicine as the proportion of glucose is high. She made analysis for me and found that proportion of glucose was 50. She said that it was low. She brought dates and juice for me, and I was able to control it and I thanked Allah. Since I suffered from the diabetes, proportion of the glucose decreased twice, one time in Ramadan in the morning while I was going to the work. I thank Allah that I continued and took dates and juice and sweet things till the proportion of the glucose increased. I thanked Allah that I felt better.

I had my breakfast and washed my face. I awakened my daughters and they sat and told me not to go to the work etc. I said that I became better thanks to Allah. I asked them not to tell me not to go to the work. I went to the work and I praised and thanked Allah. I work in the faculty of dentistry. There are Philippine nurses working with me. I sat for a while with them. They asked me why I sat here and asked me to have a rest in my office. I said to hem "no" and that I sat because I was tired. I felt afraid that the proportion of glucose may decrease and there was no one with me in the office, but praise be to Allah that I felt better.

Guest: yes … yes.

Guest: I swear by god that my daughter enlightened me more than anyone. When I sit with my colleagues, they ask me to eat sweets, one or two sweets, and tell me that eating one or two sweets doesn't damage the health. My daughter advises me strongly and supports me. She sits next to me in the events and occasions and tells me to eat little proportions of food and consider the calories in the food.

Guest: she measures the calories of the food. If the food has many calories, she brings my attention that this food contains many calories. This means that if I eat it, I have to stand up and walk till I burn the calories, but if I don't have appetite for it, it is not necessary to eat it as my health is the most important. My daughter enlightened me and I always give her now what I have appetite for it to measure its calories … she let me follow a type of diet now.

Guest: yes …. Yes, as she says that there is a type of food that increases the proportion of glucose and another type of food which requires walking after eating it to burn it quickly. This means that I eat the food containing the fewest calories because of its effect. I used to drink the tea with much sugar, but now I drink it without sugar at all.

Guest: yes, I swear by god and praise be to Allah. Since my daughter joined the nutrition faculty, she advises me. I thank Allah that I find someone who help and support and help me. I was careless, I used to drink sugar drinks and eat sweets. In the occasions, I used to eat meat and rice normally and I didn't suffer. Now, when I eat rice and meat, my daughter asks me to stand up and walk for a while, and this means that it is difficult to let the cholesterol become high and say that it is too dangerous as for your body, heart and everything.

Guest: there are types of vegetables and cucumbers. As for chicken, it is good to eat grilled chicken especially the breast of chicken. Grilling burns the fats. The persons shall use the method of grilling. You should avoid eating rice, pasta and starchy. As for the remaining foods, you have to see the menu given by the doctor. The doctor gave a menu, but openly I didn't use it.

Guest: because of my ruinous mind. I am not enlightened, Allah alone is asked for help.

Guest: yes

Guest: right

Guest: yes, I swear by god. We need more support. I can tell you that the Saudi people have many events and occasions which contain eating food. The life became hard. All the food became bad as for the health. The food put in the occasions is bad and is not healthy at all. The people put sweet and coffee full of sugar. They put many things on the banquet from all kinds of food and all of the food is starchy.

We can exclude the salads from these types of food. And now, I cannot eat the salad because of its ingredients such as sauce, mayonnaise and other ingredients. This sauce increases the glucose.

Guest: they impaired the salad also. We no longer need salad. Allah alone is asked for help.

Guest: I swear by god that I walk sometimes. I walk and become tired and can't complete because I have pain in my knees and my back. Now, it is vacation and I ask Allah for help. I sleep, eat, sleep, eat and sleep. That means that I have no time to walk. Now it is afternoon, and I shall go out for a while, but it is a short time as it is a time of having the dinner, the dinner of the seeds men. Ok, I shall walk immediately after the evening but I become dizzy and I can't continue. That's what I say to myself every day and you know that the old women can't continue its day continuously.

Guest: Yes, I need for many and many advices as even the man is becoming older and his learning becomes greater, but someone should come to enhance his awareness and we lack this awareness. Without God and the help of my daughter, I was still eating everything without warning since one year. But, God blesses my daughter, if she finds me feeling dizzy, she say to me to have some fruits in the afternoon and not to eat randomly.

She say eat this and eat this and sometimes she prepare the food for me and urges me to eat in order the level of diabetes not decreased as we have no awareness and none can answer you.

Guest: There is nothing prevents me from this except for the lost time, May God directs us all on ourselves. We cannot organize our time, if we can organize our time, we will find the time to do it, thanks for God. But, unfortunately, the time is not organized, so you feel confused all the time as we are not organizing our time and we are fault.

Guest: Yes, as it is said the brain contradicts itself. I want to make a daily schedule in order to make something in the morning, and something else in the noon and something else in the afternoon and in-between there is nothing to be done. As it is said it is dangerous to eat and sleep at night. It is supposed that you practice walking at night in order to render the calories burned before we sleep. I do not know what is meant by walking and what is the preferred time to practice it whether in the afternoon or at night? I do not know.

Guest: I liked it but after being suffered pain in my back and foot, I cannot walk; now I eat and walk for quarter an hour almost if I walk.

Guest: Yes, it is easy but this cover prevents me from doing this almost. I have a track in front of my home and I cannot walk due to this cover and I have a small entrance in which I can walk for two seconds only previously. But, nowadays I cannot walk in it as I must walk for half an hour or even an hour.

Guest: Yes, I wish there are halls for women and they must support them without fees imposed upon us. Frankly, we find them very expensive and we cannot pay these fees or the fees of joining a club, I cannot.

Guest: Yes, there is and previously I arranged with some girls to go to park in the airport as there are no men. We are arranging for two days and we are walking and there was a difference. But, when I go to work, I return on 4 and if I go for walk, I return on 5:30 and I do not find a time to relax.

There is no time to see my sons, the time is lost quickly and in the morning, we cannot go for walk because of the work. No way for walk as the system of the faculty is as the system of the hospital that is strict. I do not have a lot of work so I cannot go for walk but the female students are going in and out and we cannot move.

I wish they give us the time and the support for each other and the help to agree on walking.

Guest: Oh

Guest: No, I was never smoking and I will never smoke in Shaa Allah

Guest: I swear, they should show on me the program that they follow up. The main point is to see it, then I agree or disagree depending on whether it is suitable or not. I am old and I want someone to direct me, firstly when I was diagnosed with diabetes, none directs me and they give me the prescription of the system that I should follow up. Therefore, I follow up this system for two weeks to the extent that I feel depression, what is that? The walking sport as they never said to me anything related but they told me about the second sport for which I shall prepare myself, they never do anything for me.

Guest: Yes. This is a good program, is it right that they will apply if for us? In order to follow up and be directed especially for the patient who is diagnosed with diabetes for the first time, he shall have information. The information here does not mean I will study, but we shall have an experience, is it right or no? This means that you will not suffer as the first time you are diagnosed with diabetes.

The first time I was diagnosed with diabetes, I felt pain my chest as I lose everything, I lose the ability to eat freely, I lose the ability to have the food I bring up as this is the big fault. Then, I said I will die and I will not live for long, so I will eat. I still eat and eat without food healthy system and I have my drugs irregularly depending on my mood.

If I see that the level of the diabetes is increasing, I never eat and they never say to me that will form a danger on my kidney or something else, they say nothing. It is supposed that at the first time you are diagnosed with diabetes, the doctor shall give you instructions and advices, he shall say that the diabetes eat the body the same as Liquorice eat the palm tree. The patient shall be learned for the first time.

Guest: yes, it is supposed.

Guest: They give me the medicine after two days, they never said anything, they fasten me and I was die out of hunger, they did not give us the medicine and I should have the food they present in the hospital. I go to home feeling pain in my chest; I never eat anything except for the food of the hospital. Thus, the diabetic patient shall teach his young about the diabetes as there is a genetic factor of diabetes. You young children shall be advised not to drink soft drinks and never eat specific meals.

They shall not eat this as this kind of food causes diabetes especially if there is a genetic factor, for example my parents, my grandparents, my uncle. If I see that all my family are diagnosed with diabetes, I should make food system before I have been diagnosed with diabetes also. I was drinking sweat drinks, but nowadays, never.

Guest: Yes, I want to add that those who are diagnosed with diabetes shall be warn, they shall thank God for health, they shall thank God as they are able to walk on their legs, the diabetes is not dangerous disease but it affect the kidney and the body as whole but when you get the new awareness, you will comprehend.

You shall have the knowledge, they take me on the bed and give the medicine and they let me go out. So, I want the man to learn about it and search in the offices and centres, what shall be and what shall not be, this is good for the diabetic patient. God bless and recover all Muslims and God bless you in shaa Allah.

Guest: You are welcome.
