# Supplementary material for: Cultural adaptation of self-management of type 2 diabetes in Saudi Arabia (qualitative study)
Source: PLoS One. 2020 Jul 28;15(7):e0232904. doi: 10.1371/journal.pone.0232904 (PMC7386581; doi:10.1371/journal.pone.0232904)
Supplement: S5 File — (DOCX) [file pone.0232904.s005.docx]

Guest: I'm very keen on vegetables and fruits.

Guest: Not always.

Guest: I started to have fruits on the day when I knew that I had a simple percentage of diabetes, not before, but more than before.

Guest: Not always, sometimes I do.

Guest: Do you mean the quick meals?

Guest: Yes, I did.

Guest: Yes, I decided, but not yet.

Guest: Yes, I decided to stop having them, but I did not till now; I mean I have them but not always.

Guest: Yes, I did.

Guest: I walk.

Guest: I walk daily for half an hour.

Guest: No, after discovering that I have diabetes.

Guest: Yes, I do.

Guest: I take my instructions from the doctor.

Guest: No, I don't, because the doctor recommended the wheat bread for me, but they told me that the wheat bread increase the diabetes.

Guest: Certainly.

Guest: God's Will, it is easy.

Guest: I get help from the doctor more than my family.

Guest: Frankly, I know nothing.

Guest: I have no idea about it.

Guest: Not even a single idea.

Guest: No, thanks be to Allah, I did not feel upset or something. I did analysis at home and found a simple percentage, so I visited the clinic in order to undergo a complete analysis when they told me that the sugar level in the blood is high.

Guest: No, never, thanks be to Allah, I did not face any difficulties till now. I did not reduce or increase eating until now, it is the same.

Guest: Not completely, I mean I started to reduce the meals, not to eat too much starches and sugars, and reduced eating sweets.

Guest: What do you mean?

Guest: No, I went to the doctor who told me about eating system.

Guest: Yes, from the nutrition centre.

Guest: Yes, it is.

The Guest: Such as milk and eggs, but I don't eat all of which.

Guest: The best thing is to be free from starches.

Guest: I do not know.

Guest: No, they are not good at all.

Guest: I estimate it by the quantity.

Guest: Yes.

Guest: Yes, sure, we need more education and advices.

Guest: I don't know what advices should the diabetic follow.

Guest: Yes, I did.

Guest: Yes, only walking.

Guest: Yes, I like sports and need advices for it.

Guest: Yes.

Guest: Yes, I do.

Guest: Yes.

Guest: Yes, I practice walking outdoor.

Guest: Yes, I do.

Guest: No, I do not.

Guest: We may gather, with some friends and neighbours, and walk together.

Guest: Yes, at a given time on which they all gather and walk.

Guest: Honestly, I prefer to walk at home, because we have a big courtyard in which I can walk.

Guest: She may go to the track.

Guest: Yes, we do.

Guest: No, never, thanks be to Allah.

Guest: I don't understand, and what program do you want me to give you?

Guest: I don't know.

Guest: No, I say that I am new in this illness and I pray to Allah to cure me and all Muslim patients.

Guest: So do you.
