# Supplementary material for: Cultural adaptation of self-management of type 2 diabetes in Saudi Arabia (qualitative study)
Source: PLoS One. 2020 Jul 28;15(7):e0232904. doi: 10.1371/journal.pone.0232904 (PMC7386581; doi:10.1371/journal.pone.0232904)
Supplement: S6 File — (DOCX) [file pone.0232904.s006.docx]

Guest: No, it is not regular. I sometimes eat them according to my mood.

Guest: Not always.

Guest: I sometimes eat them if they exist, but if they are not at home, I don't.

Guest: No, I don't eat quick meals.

Guest: No, I generally don't eat them, just a little such as the grilled meat which is not a quick meal.

Guest: Continuously, I was keen on them at the beginning, but later I did not.

Guest: I don't know; perhaps because they are not always exist at home, so it is according to their availability at home.

Guest: Yes, I do practice walking.

Guest: Only once a day.

Guest: Half an hour because I suffer from roughness in the bones, so if I feel tired, I sit.

Guest: No, it is a new habit after knowing that I am a diabetic.

Guest: Frankly, it is from WhatsApp, but in the beginning, they asked me to be on a diet and on a schedule.

Guest: Not always. Some people say that its good and others say that it is bad, I don't know.

Guest: No, I don't, not always, because people are not the same, their diabetes also is not the same as everybody else, that is why I don't believe everything.The Guest: I do not work.

Guest: But I need support from the family.

Guest: No, it is not easy; I mean I can’t control it, because there are irresistible meals which may increase the diabetes level such as desserts and rice.

Guest: Yes, I did.

Guest: Such as providing diabetes analyser.

Guest: Yes.

Guest: They may help me by providing what I need, give advice regarding meals and anything like that.

Guest: All I know is that it causes pancreatic failure.

Guest: But I have no background about it more than this.

Guest: Frankly, when they told me that I have diabetes, I was a little afraid and started to avoid everything such as desserts, sugar, rice and macaroni; I really started to avoid everything, but then I could not avoid all of these things.

Guest: I felt a little upset at the beginning, but all praise be to Allah, I started to get used to it, it became something normal.

Guest: No, never, thanks be to Allah, I did not face any difficulties till now.

Guest: No, absolutely.

Guest: No, thanks be to Allah, I did not feel upset or something.

Guest: I took them from the hospital, from the health centre, from the hospital or the doctor.

Guest: No one.

Guest: They told me that the wheat bread is good for diabetes, while others said that the white bread is good.

Guest: Yes.

Guest: Such as fruits and vegetables. The doctor told me that they are good for diabetes and that I should eat less rice; I continued for a period of time but then I started to have more rice.

Guest: I don't know, perhaps because we get used to eat rice, and I personally like rice.

Guest: I need advice for sure.

Guest: Yes, I did, but only walking.

Guest: Yes, I did.

Guest: Yes, it is, but I am not good at swimming.

Guest: Yes, I do need support.

Guest: No, I don't think so.

Guest: I don't know, perhaps it is because I feel afraid when it comes to swimming that is why I don't like swimming.

Guest: Yes, I do, daily at a regular basis.

Guest: If there are directions other than my system, it is ok, I need it.

Guest: Yes, normally, and I got used to it.

Guest: No, not outdoor.

Guest: Yes, you are right.

Guest: We would like so, but everything here costs money, do you understand? We would like to walk and to go to the club, but everything is in return for money, we have not enough money.

Guest: I have nothing in my mind.

Guest: I have not any plan.

Guest: Yes.

Guest: Yes, I encourage that.

Guest: Yes, I will.

Guest: According to the circumstances, I don't know, it depends of my own circumstances.

Guest: No, God forbid.

Guest: God willing.

Guest: As I said, it should have walkways and kinetic machines for bones and diabetes.

Guest: Yes, it must have female trainers because we nothing.

Guest: Regarding the food, I have no idea.

The Guest: Well, they should put a system for us.

Guest: Well, the best thing is vegetables and fruits, types of baked meals and anything that is proper for the diabetes.

Guest: Yes, time.

Guest: Yes, I do.

Guest: Do you mean a specific time?

Guest: Yes, I think that time is important.

Guest: Because it means regular meals and regular medications.

Guest: No, I have nothing to add.

Guest: No, nothing, may Allah reward you well.
