# Supplementary material for: Cultural adaptation of self-management of type 2 diabetes in Saudi Arabia (qualitative study)
Source: PLoS One. 2020 Jul 28;15(7):e0232904. doi: 10.1371/journal.pone.0232904 (PMC7386581; doi:10.1371/journal.pone.0232904)
Supplement: S7 File — (DOCX) [file pone.0232904.s007.docx]

Guest: No, it is not regular. I sometimes eat them if they exist, but if they are not at home, I don't.

Guest: No, not always. I am only keen on what is good for me; if they increase the diabetes level, I don't eat them. I only eat what is suitable for me.

Guest: No, it is something normal.

Guest: No, I have not.

Guest: Not always, sometimes I do.

Guest: Only once a month.

Guest: No, after they told me that I'm a diabetic. So I followed a diet which requires reducing the quick meals. I eat fruits and vegetables between meals; when they told me that I'm a diabetic, I followed a diet and stopped eating these quick meals.

Guest: I used to walk for long distances, but after I had pain in my knee and back, they told me to reduce walking.

Guest: I used to walk throughout the whole week for two hours in the morning and two hours in the afternoon.

Guest: No, I used to walk with my neighbour before discovering that I have diabetes, but it is fine now and we walk to burn the sugar in the blood and have a nice talk together.

Guest: I take the directions from the patients who have diabetes for a long time and who have background regarding this illness, or from other persons older than me, such as the doctors. If I went to a doctor, I take his advice and know from him what is good and what is bad for me, what are the medications that are good for me, can I carry anything heavy? Can I walk for long or short distances? So I know everything about what fits me and what fits me not.

Guest: At first, it was easy, but now the whole family needs care, so I take care of them and they take care of me, the whole family now wants to take care of me.

Guest: My little daughter.

Guest: Because she had diabetes and followed a diet and used to walk till the diabetes level went down, not completely but almost recovered from it; however, my other daughter is not recovered, the level is getting higher than the first daughter.

Guest: In terms of what?

Guest: They say that it is the illness of the era, as almost everyone suffers from it; this is what I know about it.

Guest: It makes insulin detract from the body and cause diabetes, dizziness, headache, occasional sweating and involuntary urination; this is what I know about it.

Guest: Certainly I felt upset, but I knew about it before, because I have pregnancy diabetes.

Guest: After that, it became something normal.

Guest: Yes, I felt upset because they did not tell to follow a diet or something, and started to give me injection for a whole year. The level gets high to 300, 380 or 350, so I take injections, but after following a diet, I lost my weight and started to walk, and the level reached 200 to 180 as result. So, thanks be to Allah.

Guest: No, thanks be to Allah, I did not face any difficulties because all people I know are aware that I have diabetes and act accordingly.

Guest: There are no fears, thanks be to Allah.

Guest: From the doctor themselves, because every time I go there, there is awareness, the National Day of Diabetes; they give us papers and tell us what to eat and what not to eat, what diet I should follow, and so on.

Guest: So far, I did not follow an organized method in eating.

Guest: Grilled as they said, or bran bread, this is what they told me as they are good for diabetes; also the fruits and vegetables, fish and meat without fats, but they told me to reduce having the lean meat.

Guest: Yes, me, my daughters and parents.

Guest: I don't know if my elder child has diabetes or not, he is feeling good till now; may Allah protect us.

Guest: I tell my sisters and brothers every day that we need to have a doctor at home to teach us what to eat and what not to eat, and also what we should do and what we should not do.

Guest: 7.

Guest: They are five.

Guest: They haven't undergone any analysis or anything, so we know nothing till now.

Guest: Yes, I did.

Guest: Well, I think that we should have a special club; assigned only for diabetics, where they teach us how, when and for how long should we walk? What is the right distance? I mean to let us know about everything about it.

Guest: Yes, I do, but when I felt pain in my knee and back some day, I no longer walk for long distances.

Guest: It is Ok to me.

Guest: Yes, a club would be fine.

Guest: As I told you, they should design a track assigned only for women in order for them to feel assured and may take their children with them; in this case, I will be able to ask my neighbour to go with me and walk, something like that.

Guest: No, never, thanks be to Allah.

Guest: I inherited it from my father, may Allah have mercy on him, and our father after he retired, circumstances occurred and he got diabetes, the middle daughter was diabetic due to fear and the youngest daughter because of overweight.

Guest: The doctor.

Guest: Yes, the doctor himself.

Guest: I don't understand.

Guest: I think non-regularity cause all this mess.

Guest: Because I sleep at night, some sleep at day, there is no regularity; my children are not also regular, they don't sleep at the same time.

Guest: Because if they are organized, I will be organized also, but they don't sleep or eat at the same time.

Guest: Yes, certainly I do.

Guest: No, I have nothing to add.

Guest: No, you have said all the suggestions I know, Mashallah.

Guest: You are welcome.
