# Supplementary material for: Cultural adaptation of self-management of type 2 diabetes in Saudi Arabia (qualitative study)
Source: PLoS One. 2020 Jul 28;15(7):e0232904. doi: 10.1371/journal.pone.0232904 (PMC7386581; doi:10.1371/journal.pone.0232904)
Supplement: S8 File — (DOCX) [file pone.0232904.s008.docx]

Guest: Not much, but I do eat them every now and then but I do not show great interest in them?

Guest: I do not love many things: however, I like oranges bananas: these things, the rest means I do not love them that much

Guest: I do not eat them that is everything

Guest: Yes too much

Guest: Not much I mean once a week, or twice per month

Guest: No. However, before I used to eat them three times a week, but now only twice a month, once a time

Guest: I walk, but that is not the normal sport. However, yes I Walk

Guest: not specific times, every time I find some free time, I walk

Guest: No. Before, I walked normally, but now that they said that, I must walk whenever I have free time

Guest: The doctor, with the Dr. I means. I do anything the Dr. says, and also who answers on What's Up saying what and what not to do. Sometimes I may not be convinced, so I do the thing that I am convinced with

Guest: It is difficult sometimes. I mean: something I crave something and eat it knowing it is harmful, but it is something I desire, I cannot control it

Guest: Yes, I need it most with the family, I mean. If we hang out they always tell me what to do and not to do. They prevent me from things so I do the opposite. They say: ''Do not eat this thing'', so I eat it from being them

Guest: All of them can, but they tell me not to eat this thing: they prevent me. They can say eat small amounts not prevent it all. They should not tell me DON'T eat, DON'T do this, DON'T run.

Guest: I do not have any information but I guess

Guest: What I know is that it is a strong disease that weakens health and so on

Guest: Sometimes life sends us: I means.

Guest: No, it's ok. I mean sometimes it takes you off at once, and at once throw you to bed, sometimes it soars and sometimes it's completely steady.

Guest: in the beginning yes how they said it but later I browsed the sites to see and learn about the causes of it and what helps against it. Then it became ordinary.

Guest: Yes, I eat a lot less than them. I do not play with them. I mean playing games. I play very little as now I get fast heartbeats more often. I mean things like this

Guest: From the diabetes doctor to whom I go.

Guest: Yes. I mean, I sometimes write down some diets to show them to the doctor. He tells me what to eat and what works for me: you know I only follow the doctor

Guest: Vegetables, I mean boiled potatoes, anything boiled but when it comes to the different types I eat normally not more than 3 tablespoons, these stuff I mean

Guest: Yes, I do not need it, sometimes they sit down to eat and as soon as they finish, they say I am starting a diet then all women jump in to start too but they do not. They try in the beginning but later they eat the same old things so I sit with them and do the same thing.

Guest: Yes.

Guest: Yes.

Guest: I mean, for example, when I went to the doctor that day, he said some exercises I needed and others I do not. For example, the exercise that causes the heartbeat to increase is forbidden. So I asked for them in details. He said the most important thing now is walking then we can change it after it is easy for you. Therefore, I cannot join a club or anything like that. He banned sports for me now.

Guest: Yes.

Guest: do you mean on a daily basis?

Guest: No.

Guest: Sometimes it is due to work I go home late- so I just walk in our house, sometimes I get so tired so I sit down. It is an evening shift job. It is also not fixed.

Guest: Yes.

Guest: It is open air, in the dooryard.

Guest: Yes. Normally.

Guest: Nowadays there are no tracks in the clubs for they want them only for themselves. I do not know but I do not like and I am mainly afraid to walk alone. Sometimes I go with my sister or my mother. Sometimes they cannot hangout with me. Sometimes my father comes, as he cannot leave me alone. However, he cannot come with me, so I stay and lose enthusiasm. I stay, therefore, only in the dooryard. Therefore, women need tracks dedicated only for them.

Guest: No.

Guest: I do not understand you?

Guest: No. I mean not very.

Guest: I do not know by my God.

Guest: Yes. My sister but that she does not have all the equipment as you think.

Guest: No. whenever she is free.

Guest: Do you mean I would be forced to do things? To eat things? Sometimes, the doctor writes me food or sports I cannot eat or do. He once told me to take the stairs ten times: and in fact, I cannot even do it for once without getting tired and dizzy. I need easy and normal sport.

Guest: Sure. I need healthy food and little exercise before my night shift job. I need something to do in the morning that is different from what I would do at night.

Guest: Yes.

Guest: Yes. These days, my day is disorganized: I sleep in the morning and wake up at noon to immediately go to work. There is nothing in order.

Guest: No. They do help but I tried it once and did not benefit. I joined groups for sports as they say but you never interact with someone to be motivated.

Guest: Good and it helps better and more.

Guest: Yes. For example with changing the regime. You should do this, you should do that. I sometimes follow something and later if I go to other specialists, they always consider the existing systems as if wrong. That happens whenever the doctor is changed. They should start with only one.

Guest: I shall talk to them about my experience and what happened with me. I mean to say that you have done so and so: and also show them how I lost weight, how I dieted, how my health is better and how I would want them to join.

Guest: what do you mean?

Guest: Yes.

Guest: I mean it is possible.

Guest: Yes. It encourages a lot but you would also find dishes you may like because of its appearance, but when you taste it, I do not know.

Guest: God willing maybe.

Guest: I do not want people to use it against us. Whenever I do something, they make me afraid of it saying it causes diabetes: it raises its levels. This is the most important thing. I do not want them threatening me all the time reminding me of that who lost a leg because of diabetes and that who had it broken and that whose limp was cut because of it. This is the most important thing.

Guest: You are most welcome.
