# Supplementary material for: Cultural adaptation of self-management of type 2 diabetes in Saudi Arabia (qualitative study)
Source: PLoS One. 2020 Jul 28;15(7):e0232904. doi: 10.1371/journal.pone.0232904 (PMC7386581; doi:10.1371/journal.pone.0232904)
Supplement: S9 File — (DOCX) [file pone.0232904.s009.docx]

Guest: God bless you, Thanks God

Guest: actually, the first diagnosis was on 1428, approximately on 2007, 2008, almost since 11 years.

Guest: one day by coincidence when I felt something wrong and as my father suffered diabetes as well, thus he played a vital role in informing me that the symptoms that I already suffered are the symptoms of diabetes.

He has Diabetes Detector at that moment and it was a home detection, then I was performed Blood Test and I found that the diabetes rate was higher than 300 nearly on this day. I felt thirst actually and you know that the diabetes patient is drinking water but he is not watered as well as he always suffers perspiration as well confusion.

Guest: There is no doubt that when you feel such, you will be shocked. He was expecting this due to the family history with this disease, the father, the mother and the grandparents, thus he was expecting this and he was waiting for this. But, I was surprised as I was young, I was 28 or 29 nearly. Thus, there was a surprise despite he was waiting for this.

Guest: Unfortunately, we confess that we have bad food habits, very bad ones. My relation with fruits and vegetables was not so good really especially the fruits, Is this a food routine to which I used!! Is this a social behaviour!! I do not know but my relation with the fruits still very superficial.

Guest: no, Thanks God, they are available but there is a self-insufficiency on my part. The fruits are found before me but I am not keen on having them. For sorry, I was very shortened and if necessary, I am keen on specific types due to their usefulness. When I was having fruits, I say oh my god I want a specific vitamin or specific fibres and I was not having them as sweets. But, the fruits which I am considering as sweets are the grape and pomegranate as I like their taste and not having them for their usefulness as I have not a desire to the fruits.

Guest: nearly, I prefer the grape personally, the pomegranate, the watermelon, pineapple and crazy, I know that they increase the diabetes but I eat them in few quantities and sometimes a season passes through and I do not eat the pineapple and sometimes I eat it for two or three days during the season that lasts for three months. Moreover, in the season of grape, I eat it twice yearly.

Guest: No. in fact, leafs with the meals, meaning that I prefer to eat leafs alone as they are very useful. But, there is no doubt that I prefer it mixing with the food, for example if the meal consists of rice, I prefer to eat leafs with it. In addition, as well as the salads, I like to have as it is very useful and it makes me feel relaxation.

I never deny that I feel relaxation when I eat salad, leaf and the green vegetables and I feel that they do not increase the diabetes. Regarding the fast food, I eat it in few quantities and I never have the desire and not keen on have it but sometimes I am forced to have it when I am traveling, thus I ask for a fast food with soft drinks. But, unfortunately after having this meal, I feel that the diabetes is increased on contrary to having leafs or specific food.

Guest: I swear, I have it in very few times and sometime two or three month have passed through and I do not have fast food.

Guest: For example, Pepsi or Cole or the soft drinks in general, I do not drink them for three or four months.

Guest: Yes, Yes I tell you this with self-satisfaction as I do not have the soft drink except with these meals. Personally, I do not prefer these meals but when I travel with my family, sometimes the children are forced to have it, but when we are going for shopping, I do not prefer this and if it is suitable for me to go shop or market or even minimarket, I do this to have a yogurt and piece of bread to have them rather than the fast food. Psychologically, this is due to the diabetes and even before I suffer this disease, this was my habit; I was not keen on having fast food such as burger or other things.

I tell you that I have them when I am forced to do this and in limits as it can also cause me surfeit and I do not know if there is a problem in my body or there is other reason. But, before I suffer diabetes, if I have such fast food, I suffer psychologically and ventrally.

Guest: I swear, I have it in small quantities even before I am diagnosed with diabetes and know this. I used to have fast food in few quantities. But after I have been diagnosed with diabetes, the space between me and the fast food is increased. I am not care for having it ultimately as I said before, I have not the desire to have it. In addition, when I remember that the soft drinks accompanying these meals increase the level of diabetes, I try to get away from them as much as possible.

Guest: I swear, regarding the sport, I never practice sports only walking. I never stop walking to the extent that when I stop walking, I suffer psychologically as when I practice walking, I feel more comfortable psychologically but I do not know if the reason behind this is the diabetes or not.

When o practice sport especially walking, this is reflected on my psychological state. So, I am keen on practicing it continuously. In fact, I never adhere to walk for neither specific spaces nor specific time and never adhere to walk daily, but weekly, I used to walk for 5 days.

Guest: Sporting activity? No, I do not.

Guest: No, I practice only walking and I do not practice it in specific time but if there is an appropriate time when I back to home. If I wear sportswear, I can run but not for long space in order not to feel tired but I have no other sporting activity.

When I travel, I walk a lot for long spaces nearly 6 or 7 K and more but only during traveling. The matter is different when I walk a lot; I feel improvement to the extent that I feel the desire to have sweets and snacks during walking.

This is the case when I walk along more than 6 or 7 k for more hours, so I feel that I want to eat these things in addition to ice cream. I used to have ice cream for only one or two time during the year in maximum and I used to have this when I travel as I feel the desire to have sweets when I am on long distances whether in travelling or during walking.

Guest: No, in fact I did not practice walking formally before I suffered diabetes and even I am diagnosed with diabetes, I seat for 6 or 7 years without practicing walking, on contrary I never walk as result of my work conditions as I was working in a private sector. This is the reason actually but I used to exert an effort but not sporting one.

There is a physical effort but not sporting one. I do not know if this would benefit in diabetes or not. But, after I first diagnosed with diabetes with nearly 6 or 7 years, the walking sport becomes my friend.

Guest: may be, I cannot assure this, why? As if I do not walk for two days, this will have a bad impact on my psychological state and I feel relaxed and in good psychological state if I walk.

Guest: I will not hide, as a family, we have diabetes culture actually, the young as well as the old, the male and female know about it, but why? As many of us are suffering diabetes, starting from the grandparents who were suffering also diabetes, then our parents on the level of brothers and sisters, the niece, the cousins and nephews.

As result, diabetes disease becomes subjects which are handled daily in our sessions from one hour to two hours. This disease must be tackled by us as a family as well as our relatives, we have many information about diabetes even if we do not read about in in references nor the Holy Quran, Do you know how? Because of the smart systems and devices as well as the social media, we are now having many resources.

Really, we may not be accurate that is supposed to be found and searched by us, but the subject of diabetes become our daily subject that we are deliberating, we have these information not from the programs like Instagram or the message through what's up nor Facebook, but I get such information from my friend who get a piece of information somewhere as well and my brother who gets a piece of information somewhere else.

My father also get an information as well as my mother read about it, Form where? I do not know but they have a good experience in diabetes. When I speak to you about me personally, I rarely visit diabetes centre or the endocrine centre to check about diabetes. I do check my diabetes level by my own, I have the detector and I evaluate the diabetes level according to my estimation. The last time I visit the doctor asking for his advice regarding diabetes, was before 6 years.

After this I do not visit the doctor to ask him about the diabetes, I depend on my estimation, I know now the food that increase diabetes level and the food that do not affect its level. As I said before, this traces back to the experience I get from my grandfather, grandmother, my aunts, my uncles, my father and my mother. Thus, I get this experience.

Guest: I get the drugs from the city clinic if the drugs that I have are available as I get it free of charge. In case they are not found, I get it from the pharmacy.

Guest: I came to do diabetes analysis that is called 6-moonths analysis but why are they calling it as such? I do not know.

Guest: Yes, the accumulative, I do this for check-up only.

Guest: I swear, if I say it is easy, it would not be in fact, and if I say it is abstained, it would not be as well, but it is abstained easy in fact. Sometimes. Medically you are prevented to do something’s but as the one wants to break down the routine sometimes; he does not prevent his himself in order not to affect his psychological state. But, we shall not exceed the limit and this is the easy way, thanks for God.

If I have one dates, I do not eat all the quantity, I try only to have two or three dates and if I have it, I have them in an occasion or ceremony only. In addition, if I taste the sweet, I try to punish myself be depriving myself from having things that I used to have on daily basis. I try to replace this by that so I should follow these limits.

For example, when a dish of sweets are presented to me, I prevent myself from having it and if I am forced to eat from it, I eat with limits meaning that to share others only not to eat for eating itself.

Interlocutor: Ok. Do you need any help from anyone, For example, a support from a family or encouragement as setting a specific food program or any support in terms of tools or materials that you are in need?

Guest: I see that it depends on the age of the patient, if we say that those who suffer this disease are ranging between 25, 50 and 60 years old. I see that those who are in earlier age, do not need the help of others, they serve themselves and it depends on the person himself. But, there is no doubt that the family has a big role in supporting and increasing the morals.

All these matters make us feel serenity and when the man's problems are decreased by the society around him in terms of his family, sons and wife who are getting the problems away from him; this will be reflected on decreasing the level of diabetes. But, there are matters that happen unwillingly, but if we ask how many do they happen?

In addition, the accurate diabetes detector that the patient need shall be saved greatly, moreover the medicines shall be saved in the healthy centres, all these things are needed by the patient, other than these and in the age of 40, the same as me, I do not need for anything, thanks God.

Guest: My information about diabetes: Diabetes is a disease or let’s say it might be a disease with which I deal. I have sons, one of them is suffering Bean Anaemia, so I do not consider it as a disease but I do not know what is its medical name, but it is the disease that affect and hurt specific system in which there is an inflammation and you should take medicine for specific time to be recovered and this is called the disease. On contrary, this is not considered a disease rather it is a symptom or an indication against which the patient shall be protected. But, what is its name medically? I do not know, it is merely a subject against which the one shall be protected.

If you follow up a full integrated protective program, it becomes an indication or emergence not a disease but it will last forever. If you neglect the protective system against this thing, in this case, you will affect yourself. If you consider the diabetes, as supposed, as friend, this will be reflected on you neatly.

But why, as it prevents you from the bad way of eating, it forces you to practice sport and other practices that will benefit you personally and if you consider it as an enemy and you forget about the protective program, it will destroy you.

Guest: I swear, there is no subject that is void of challenges but if the human is well and merciful, he will be offered all tasty dishes and as you know in this time the offer is plentiful, thank God, and the self is furious due to the things that are offered and cannot be prevented. This is considered as the greatest challenge that face the person. But, we as Muslims, are grateful for God as when we are deprived of something, we are rewarded by God, thus this is considered as our consolation against this challenge firstly. Secondly, sometimes, you feel frightened if you are travelling or going on a trip and you need to exert an effort. You will have the medicine with specific dosage, but you feel fear of the decreasing level, and this, as being diabetes patients, is the most difficult matter that we passes through as its results are very tiring and exhausting and this is considered the most frightened element against which we fear due to the decreasing level on contrary to the decreasing level of diabetes. Decreasing the level of diabetes are accelerating always and if you are not ready for this decrease by having the precautions such as sweets or juices, you will realize the matter but sometimes you are alone, thus it will be difficult for you to make the first aids to avoid such thing and this is the most exhausting subject from which we suffer.

Guest: in fact, Diabetes centre in King Fahd Specialized Hospital, God reward them, and the responsible are giving as private booklets with a specific food system for the diabetes patient and one for those who suffer the blood pressure. As I told you we are not ideal and we are not caring about these things.

But, as I told you before the medicine subject, we are following through experience, we know things that raise the level of diabetes and we know things that decrease it, it is through an experience not the papers.

Or can we say it is the traditional meals, such as rice and meat, I mean El Kabsa, and do you have it and calculate for its calories?

Guest: Yes, I used to have the breakfast, but since ten years, I used to have only two meals daily and I know that the diabetes patients are in need for more meals meaning that he should multiply the meals but decrease the food he eat. In fact, I cannot do this as result of my work and my daily program, I cannot do this.

10 years ago, I used to have two meals, the breakfast and the launch, if the launch is delayed, I have the dinner and the breakfast was in small quantities but anyway I have two meals only.

Guest: I think that there is no doubt that the food programs are useful and if you help your family with right food program that is comprehensive, they will eat great number of various foods in terms of vegetables and fruits as well as the meats. There is no doubt that these are considered as food supplements but we should adhere to them, but we as a society are finding difficulties in considering these matters.

In my opinion, diabetes disease is a genetic disease and if we follow specific healthy food program, we will not suffer it. Diabetes disease as a genetic disease will be suffered inevitably, whether you vary in your food or not but anyway the variety is useful whether you suffer diabetes or not.

Guest: in fact and there is no doubt that there is an ideal time for practicing sport, after eating in specific time or before eating, in the morning or at night. Sometimes, the healthy sport need for specific time in order to achieve the best results. But, unfortunately, I do not know what those appropriate times are if there are scientific or medical known times.

I do practice sport according to my conditions, sometimes in my spare time, some times during in the afternoon. In addition, I know that the best time to practice sport is at night in late time, there is no doubt that nothing is more preferable than sleeping after practicing sport, but it depends on your condition.

Guest: the time and the lack of encouragers, sport shall be collective work as groups, this is the preferable but on the individual level, I do not like this. The subject of joining sporting club is considered difficult for me as I do not like it and I do not think in making sporting room at my home that contains special equipment in full to practice sport and I will not adhere to do this.

Guest: it does not fit me, as a societal habits and traditions. The clubs means to adhere to specific time or hours in accordance with the trainers and their time and I cannot subject to this personally. As an employer, I have specific working hours and I can join the club at night and the train can organize the time but I cannot adhere to this.

Guest: I like walking, walking is useful, I have a farm, when I go there, I practice walking and sporting movements other that walking during work. But. In the city, I have not specific place to walk in any place, I walk in malls, you know why?

As malls are considered as living roads where there are noises, activities and movements, these places such as Sokar, city centre. When I walk in these places, I never feel time and I can walk for long distance and this is the advantage.

Guest: Yes

Guest: I never take this subject and stop smoking seriously and up till now, I do not hate the smoke. Those who stop smoking hate the smoke but I do not reach this phase on contrary I like smoking frankly.

Guest: If I hate smoking, I never need a help and I will stop smoking.

Guest: do you mean at this moment or it will last forever? What do you mean by the form of the program? Is this program is temporary or permanent or how?

Guest: really, it is good idea in its own. I do not know how it will be applied or performed. But, I suggest establishing healthy centres in each district as well as diabetes centres, why? As diabetes nowadays becomes part of the community, it is the companion. In fact, the health clinics have no possibilities that serve diabetes patient.

I think that diabetes is in need for private centre and the same centre of diabetes shall be found in each district in which there is a health food department such as buffet for diabetes patient at least in order to learn how to get the culture, how to talk. Previously, I did not enter or eat from it, but they talk about this healthy restaurant.

Let us take an idea from them or their food for example as in these centres, we will know about specific food as there might be a tasty food and at the same time it does not increase the level of diabetes. These centres are controlled over by males and females as well and these centres shall be divided into male and female department as well as the buffet.

These centres also shall have sporting hall and the diabetes patient shall practice the sport that is appropriate for him such as swimming as well as I prefer that this sport to be collective not individual one in order not to be difficult to be practiced.

Guest: If we say that it takes the form of an application, I think it will be effective and if we say it takes the form of a message or warn and there will be a call centre and operations centre that will give you the daily updates such as the availability of certain drug, the dosage, it will be possible to find that information that will need for a challenge. For example, there are new drugs that are produced such as the pancreatic stimuli, so we shall consider this as we never hear about it, its production, its dosages or the drug. Thus, if these applications are available, we will know information about this drug that is discovered lately and without this means, we will lose a lot of information. Thus, if there is a centre that notifies us through a message or a phone call, we will know that it is available in the healthy centres or it is available in the treatment market, its replacement with less dosage. This will be the best, thus the application will be effective.

Guest: Yes, Yes, there is no doubt that this will be the best and it’s my personal sense that it will be good and have good results in Shaa Allah.

Guest: No, In fact, I have no addition. I want to thank you for your hosting and taking the information. No doubt, we utilize from you and I wish at the end of this interview to add and utilize you and our experience will help you in developing your programs. As well as, we want to say that in Shaa Allah, the future will be promising and we wish a new surprise will happen in Shaa Allah by your hand and as result of your efforts and we wish that God will promote and evaluate your study in Shaa Allah.
