# Supplementary material for: Cultural adaptation of self-management of type 2 diabetes in Saudi Arabia (qualitative study)
Source: PLoS One. 2020 Jul 28;15(7):e0232904. doi: 10.1371/journal.pone.0232904 (PMC7386581; doi:10.1371/journal.pone.0232904)
Supplement: S10 File — (DOCX) [file pone.0232904.s010.docx]

Guest: Peace be upon you, My brother, regarding the food system, this system is depending on proteins, vegetables and some fruits.

Guest: Yes

Guest: Never, I do not eat ultimately.

Guest: No, after being diagnosed with diabetes.

Guest: Yes, I was eating fast food but sometimes not usually.

Guest: Two or three times monthly.

Guest: Yes, I walk daily for half an hour.

Guest: Almost, 5 times in a week for 30 minutes permanently.

Guest: No, after being diagnosed with diabetes.

Guest: Yes.

Guest: I swear, I have got some of these sources from the nutrition clinic at Diabetes Centre here, the other are got from Internet and through those who have experience as they are diagnosed with diabetes since many years, from this and there.

Guest: It is not difficult and it is not easy as well, it lies in-between.

Guest: To control over diabetes with 100% is very difficult but caring about it and never neglecting it is easy.

Guest: Sure, family support is essential, it may not be by the appropriate form and amount, but it is found.

Guest: at the first position, the wife, the second position is my friends whom I see daily.

Guest: I know that it is a disease, if you respect it and its habits, it will be as a friend and if you do not and never care about it, it will destroy you.

Guest: My feeling not is differing from the feeling at the beginning of the diagnosis. My feeling now is coping with diabetes and I never felt anything different. I feel that I am natural person. But at the beginning of the diagnosis, there was a shock and I was denying this but with time, this vision is changed.

Guest: Yes, there are some challenges that face me during travelling, the non-availability of the appropriate food even in the restaurants. Sometimes, there are no restaurants for diabetes patients. In the super markets, there are no places for diabetic patients or even goods of accommodations for them.

Guest: never, There is any difficulty socially.

Guest: At the first position, I get the advice from the nutrition specialist at the Diabetes Centre, at the second position from the physicians and the consultants of public health, whether through the social media or private centres.

Guest: No, often through the types of food, it shall be divided into sew quantities of carbohydrates, few quantities of proteins, a lot of fruits and vegetables as well as water in big quantities as well as the meals containing water.

Guest: Yes, for sure, sometimes there is ignorance on the part of the family and on the part of the diabetes itself and on the part of myself, what is the appropriate food that is convenient to the diabetic patient and non-diabetic patient when the family is met together on one meal.

Guest: The physical exercises, I never do and I wish I could get the advices of the physical exercises especially body building.

Guest: Yes, swimming, but any other types of sport no, I never felt afraid of infection but there is no opportunity.

Guest: No, I am not regular but I walk for 30 minutes daily but not at the same time not hour, but in a daily basis regularly.

Guest: Sure, of course and especially the time and when I can practice it, sometimes, the best time to practice sport is before the main meal or after the main meal? How long and how many time in a week? I want to know such things.

Guest: Yes, Yes in the open air.

Guest: No, I am not smoking.

Guest: No, I think that the application through the smart advice is preferred as you will get it all the time, any time and when you want. If you forget something, you can return to it secondly, the phone calls are limited to specific time and specific domain.

Guest: Do you mean the sporting one?

Guest: I want you to mention more details? Regarding the form of the program that I need today is the program of thin as I suffered being very thin and I want to know the food system that will build my body and that is related to the sporting system, I mean body building. Now, I am in a dire need for foods that build body and provide it with energy and power but they do not increase the level of diabetes.

Guest: The Schedule you mean that I follow it now.

Guest: It may contain updates.

Guest: My morning schedule is to have the breakfast that consists of one piece of bread, boiled egg and I wish I could not increase these items in order not to suffer problems in regards it diabetes. But, I can increase other dish such the vegetables or fruits, in the afternoon, I have fruits not a full meal and at night I have the main meal.

Guest: in the afternoon, I do exercises.

Guest: Yes and I walk at night.

Guest: Yes

Guest: No, before I was interesting in.

Guest: Yes, as they are the only natural sources of sugar that will last for me after being diagnosed with diabetes as well as following the advices of the nutrition specialist to have fruits increasingly.

Guest: No, Never.

Guest: If the diabetic patient is persuaded by his affection, if he neglects himself and the level of diabetes, after 12-15 years, he will find himself losing the most vital functions such as his sight, kidney and his tissues, the blood vessels, the cardio diseases, but If he preserves himself and the level of diabetes as well as the food, I expect that it will be a normal disease like flue and it will not harm human or anyone, this is the internal persuasion of me.

Guest: yes I am looking forward to care about the diabetic patient not the diabetes as a disease. I want in case I go to the airport, I find a place for the diabetics, if I go to restaurants, the hotels, I will find a place for the diabetics, even inside the airplanes, there would be a place for the diabetics as it is considered as a popular disease and there are more and more diabetics.

Guest: I mean to find meals in the airplanes.

Guest: This will be appropriate for the diabetics, in the airport, there should be part in the restaurants to present meals for the diabetics as we are forced to have inappropriate meals or never eat.

Guest: There are tracks in the districts and special places for walking that is good and in the open air that is appropriate for walking.

Guest: yes, sometime it is very cold, dusty or airy, sometimes it is very hot in the summer and all there are considered as obstacles. Thus, at night it is very suitable.

Guest: Yes

Guest: I wish there are first-aid centres in which there are nutrition specialist as he/she will be easier and more approaching that the nutrition specialist found in diabetes centre due to the over load over the diabetes centre whereas in the healthy centres, there is no load and the communication will be easy and on a monthly basis.

Guest: Through my experience, regarding the food, I know that there are some kinds of food that increase the diabetes and sometime due to the tension or the psychological exhaustion. But, after the suffering of diabetes for one year or less or more than this, the diabetics realize the foods that are appropriate, realize what can increase or decrease the level of diabetes' level.

Guest: Yes, Of course I have a detector and I follow up the analysis when fasting and after the main dishes, even the random analysis, sometime I do it. In regards to the advices, I resort to the physician of the centre in the district periodically asking him for advice. Regarding the medicine, I have them regularly.

Guest: No, there is not except what I have said, I think that I say everything.

Guest: You are welcome.
