# Supplementary material for: Cultural adaptation of self-management of type 2 diabetes in Saudi Arabia (qualitative study)
Source: PLoS One. 2020 Jul 28;15(7):e0232904. doi: 10.1371/journal.pone.0232904 (PMC7386581; doi:10.1371/journal.pone.0232904)
Supplement: S11 File — (DOCX) [file pone.0232904.s011.docx]

Guest: Peace be upon you.

Guest: I swear, it is a natural consumption not an excessive one.

Guest: No, not permanently

Guest: No, never I do not have it, very few.

Guest: Yes, I was eating fast food.

Guest: Approximately two to three times weekly.

Guest: No, I was a football player previously and I stop it since more than 15 years.

Guest: I swear at the beginning, any man hears this, he will be shocked and will decrease the food he has out of fear, then he starts practicing the sport. But, quickly he returns to the natural situation and he begins to eat everything like sweets and all items.

Guest: due to my work at the Ministry of Health and my major as a pharmacist, I have sufficient information about diabetes, wide information. In fact, at the beginning, I did the analysis to know the rate of the diabetes around 4:5 times daily. Thus, I was decreasing the calories I had as well as sugars and fats. I was practicing sport then I have the drugs regularly.

Guest: I have many sources that I got and I try to read anything about diabetes. There are always lecturers and seminars about diabetes and I attend them continuously.

Guest: due to our nature here, there is difficulty as sometimes you wish to organize your food but gathering with the colleagues, the food habits and orders that we make, those are things that limit organization and regularity. So, there are sever difficulties that we suffer.

Guest: There is no doubt that you are in need for this help and in case there is no help, you will fail in making food system. Therefore, every diabetic patient is need for a help.

Guest: The most approaching person like my wife and those who are existing with you at home, they will help us in making and organizing food, its order and the type of the food. Those are number one as well as following up the physician and it is very important.

Guest: I know that diabetes is spreading very quickly and man coexist with it. but, it is considered as dangerous disease if he neglect himself as it destroys all systems in the body in terms of teeth, sight…etc.

Guest: I swear, you will enter within psychological states and you will always think in the peoples who are suffering foot amputation, the peoples who lose their sight as result of suffering from diabetes as well as thinking in those who suffer kidney failure. All these diseases are resulting from diabetes and neglecting it.

Guest: Sure, there are. For me, the overweight due to the genetic factor is not found nearly. The difficulties appear in organizing food, practicing sport, the difficulties in our habits and traditions in the society, as well as relaxation and food. All these difficulties are suffered by diabetic patient.

Guest: Due to the nature of my work at the Ministry of Health, all surrounding colleagues are having information through the nutrition specialists, thus I got advice and consultation from then continuously.

Guest: Through the calories, when firstly diagnosed, we have a strong reaction, we avoid sugars, fats and this lasts with the patients due to their awareness and because the habits that we have as I said before in terms of nutrition at home sometimes and this will help us and these are the challenges that face all patients.

Guest: Yes, there is need for this.

Guest: Yes, I do practice a physical activity that I never stop practicing sport for more than one week or 1 days at most.

Guest: Walking sport only and sometimes swimming.

Guest: Yes, I need for this advice actually and despite I know and realize it but I need for reminder.

Guest: No, I am not regular.

Guest: Due to not managing or organizing the time in away that is regular and written due to the family conditions sometimes, the social conditions that prevent you from practicing sport.

Guest: No, I need only for will and planning in fact. There is no doubt that the conditions can prevent you from practicing sport due to the surrounding atmosphere, but you should establish a room at home to practice sport.

Guest: No, for me it is easy, there are no difficulties.

Guest: I swear, there is no doubt that due to the overweight, this affect me as I suffer an attack if the weather is not pure, this I cannot walk.

Guest: Yes I like it.

Guest: No, I never smoking

Guest: I swear, this programs are required but sometimes we face difficulty in the process of transformation and in fact I have a proposition, in the governmental facilities such as hospitals as well as the specialized hospitals, the employers shall take half an hour daily to explain the program of sport, the program of nutrition that would be better no doubt. Sometimes, you are planning for these programs but quickly you break them down due to the weak will.

Guest: Yes

Guest: I swear, there is no doubt that if there is a connection with the district and what is called Family Medicine, he will be the family physician, the district centre is the family physician. If there is a linkage, it will be strong and best. To make a program on your device is the best connection.

Guest: The routine

Guest: there is no doubt that practicing sport in fact on continuous basis is benefiting and I am aware of the process of diabetes. I note that when I make special program of food with practicing sport, the rate of diabetes is natural. But, if this is not regular according to a schedule, you will forget and practice very bad nutrition habits. You will find that the diabetes level is increased seriously. Thus we lack organization, the process of continuity, the process of putting schedule for food, the breakfast, the launch and the dinner. So, there would be a variety and it will affect you.

Guest: in fact, as I said I want only to establish centres at the same time there are many centres for sport and practicing it. But the peoples have barriers due to the process of participation due to the increasing prices sometimes. If there are specialized centres within the governmental circles or inside the districts in which there are supervisions on sport practicing whether for men or women, this will cost them very few amount or even for free. This will enhance the awareness of the people and they will practice sport continuously and will organize their food system.

Guest: God bless you
