# Supplementary material for: Cultural adaptation of self-management of type 2 diabetes in Saudi Arabia (qualitative study)
Source: PLoS One. 2020 Jul 28;15(7):e0232904. doi: 10.1371/journal.pone.0232904 (PMC7386581; doi:10.1371/journal.pone.0232904)
Supplement: S12 File — (DOCX) [file pone.0232904.s012.docx]

Guest: Allah blesses you

Guest: We eat fruits and vegetables almost daily.

Guest: Yes.

Guest: No.

Guest: Yes.

Guest: Fast food is a cause of diabetes, it contains oils and fats. I eat only at home.

Guest: Yes, a little.

Guest: Yes, I work with my hands; I work and rest, walk and eat. I avoid food that contains sugar or fats.

Guest: The nature of my job is doing everything with a rest. I do everything, I have no worker or employee, I decorate everything with my hands.

Guest: I'm a farmer. I work -thanks God- with my own hands, I mean I make a lot of movement daily.

Guest: Yes, almost daily.

Guest: No, before I got diabetes, now I'm 12 or 13 years old with diabetes.

Guest: Diabetes information is well known, simply avoid the fast fats.

Guest: This is the important thing: dinner should be a light meal and have this meal then you go to sleep.

Guest: Fine.

Guest: Yes.

Guest: The sources of palm trees are date palms; it is taken by my father. My father was a farmer and knew sufficient information about palm trees.

Guest: Right.

Guest: OK.

Guest: Diabetes is not a new disease; its instructions are well known. If you go to the diabetes centre, you will get instructions and information. If you use the internet, you will get instructions... it is not a new issue.

Guest: It is old issue and the patients are growing because of food.

Guest: From the centre, the doctor tells me certain matters that increase diabetes.

Guest: No, it is not difficult, you should avoid certain things.

Guest: About what exactly?

Guest: In the first place, you should do not eat and saturate, because most of the time we have no will and consume fats and such thing is harmful and raises diabetes. Your dinner should be yoghurt, brown bread or fruits, because you saturate and it is useful having no harm.

Guest: Yes, married.

Guest: Yes, she must help; she is the mother of children and the main pillar of the house. She is the one who cooks and decorates. She knows the nature of your food and drink and she is aware that it must be free of fats and sugar, like the brown bread (bread of diabetes and diet).

Guest: Yes, she prepares it and all other necessary food like low- fat yogurt.

Guest: I know that diabetes disables the pancreas function which means that it will not function and work with a 100% capacity, it will be about 50% and it does not stop.

Guest: The first feeling is tension, anxiety but later on you got familiar with it. It will last with you the rest of your life.

Guest: No, there are no difficulties. Everything is prepared for me and the house has a garden.

Guest: By my doctor.

Guest: Yes, the doctor referred me to a nutritionist to teach you what increases diabetes, you can eat this and it doesn't raise blood sugar. You can benefit from it but we don't trust 100%, so we apply about 50% because it is difficult to apply.

Guest: As we said brown bread, low- fat yogurt and other stuff containing low sugar, white bread for example would trigger blood sugar.

Guest: In fact, I would like to do such things but you have not to eat heavily.

Guest: Yes, it is necessary. Every diabetes patient needs someone to give him advice, anything from which he can benefit.

Guest: No, I just walk. I walk daily about two or three kilos.

Guest: I mean every two days.

Guest: There is no certain or exact time for it. When I'm free, I walk.

Guest: According to my circumstances, in some days the circumstances and times allow me to walk.

Guest: I walk only at night.

Guest: Yes, the weather.

Guest: In fact, we don't follow the guidance 100%, we apply only what is useful.

Guest: Yes, I walk everywhere.

Guest: Yes, walking is important; it is my favourite.

Guest: In fact, anyone who doesn't walk becomes lazy. It is very simple; if you walk for one or two days you get familiar with it.

Guest: Yes.

Guest: No, Except by the will of God Almighty.

Guest: In fact, I think Allah only can help me right now.

Guest: No, I don't need help. It is Allah's willing. I heard about certain medicine and pills but I don't want to use it.

Guest: Great.

Guest: I want sports only because it is the enemy of. The enemy of diabetes is sports. When you practice sport, blood sugar will decrease and when you stop practicing your blood sugar will increase.

Guest: I prefer its way to be walking or swimming in order to decrease blood sugar.

Guest: No, I have nothing to add.

Guest: In fact, those have diabetes now should avoid fats, desserts, eating and sleeping. When you eat and sleep directly, you can get diabetes easily.

Guest: May God bless you.
