# Supplementary material for: Cultural adaptation of self-management of type 2 diabetes in Saudi Arabia (qualitative study)
Source: PLoS One. 2020 Jul 28;15(7):e0232904. doi: 10.1371/journal.pone.0232904 (PMC7386581; doi:10.1371/journal.pone.0232904)
Supplement: S13 File — (DOCX) [file pone.0232904.s013.docx]

Guest: I consume fruits and vegetables after breakfast exactly at 12 pm, I eat from 12 to 12:30 green leaves, like arugula, parsley, cabbage and lettuce, besides, and when I finish them, I eat half of an apple or an orange and a kiwi that is my daily consumption of fruits for 20 years.

Guest: Guest: Yes, I care about my sons and myself too.

Guest: No, before, a long time ago before diagnosis. That is a lifestyle.

Guest: No.

Guest: No, I actually do not like restaurants since a long time ago. I sometimes need the original pizza, those of big restaurants’ names.

Guest: Yes, I walk like half a kilometre a day, sometimes for a day or two no,, I usually walk.

Guest: No, no it is old.

Guest: No, no I do not believe in Arabs’ treatments at all. I do not admit it.

Guest: The source for diabetes centre, I do not believe in anything else.

Guest: No, no, no sometimes a lot of them are not right.

Guest: Yes, it is, I check for 4 or 5 times daily.

Guest: I’m careful, I am keen on healthy food, eating meat: meat increases sugar in some way normal, especially meat, but if you eat, for example, rice, soup or vegetables, like broth, they would pose no problem. However, meat increase diabetes fast.

Guest: No, my wife knows my system herself and if I am invited anywhere I am trying to eat salad or light food, I am not interested in eating meat.

Guest: I know it is a disease, through which I should follow a regular treatment. My relative was a diabetic patient for 50 years, he died now, God bless him, he did not die because of diabetes although he was diabetic for 50 years, and it was his destiny as he was following the treatment.

Guest: No, no when I was diagnosed diabetic I used to drink big quantities of water excessively. I might have drunk 4 to 5 litters a day. When they told me “you are diabetic”, I received it with satisfaction because my parents and 6 siblings were all diabetics: the 5 were diabetic.

Guest: From my viewpoint, I always do the checks. That means that if I felt anything, I would check immediately. I eat something like biscuit or eat anything that increases its level a little and ask my sons for making anything light. However, the most important thing for me is wearing shoes; I do not fear anything except legs because I am afraid of gangrene.

Guest: From the centre, everyone gives you something that they do not know about, but the doctor is for sure right 100%.

Guest: The first thing is fruit, in addition green leaves leafs, this is excellent. Put fats get away from meat, and if you want to eat meats , put it in the oven till fats dry out like roasting or grilling that has a little effect and do not always eat meat or chicken but fish is good.

Guest: Normal, normal, no I do not have any problems.

Guest: No, no.

Guest: No, 20 years is not a short time, I depend on myself, I got used not to eat anything like sweets or eat 10 mm 15 mm 20 mm if I want, I mean only simple things. Sweets are a great problem and sugar too. I think I did not drink soft drinks for more than 10 years and processed milks too. However, it is normal to drink milk and maybe 2 or 3 cups a day but I do not acknowledge Almarai.

Guest: No, not regularly.

Guest: I have a gym at home, so I can lift weights. I mean I exercise every day. So see the belly if I do not exercise you will see a big belly, I mean I exercise at home, I have a private room.

Guest: No. No. really I do not, sometimes I do not practice for two weeks or a week, but I train the belly.

Guest: It depends on the psychological state sometimes I became tired, that is the reason.

Guest: No, no there is nothing preventing me, because I have a bed there, I mean I sleep at this room in the morning, I do not get out of my private room.

Guest: Right now, my body is excellent; I know that weight will be right.

Guest: I do not need anything, I mean my weight is not great, I do not have a belly and I am not so fat, I depend on myself I know that I am doing right.

Guest: Yes.

Guest: It is ok, within every two weeks. I have a pool here. I swim.

Guest: Tracks. The track is excellent, like after the evening prayer, it is excellent, but tracks are not here, and if the track is here, maybe I will not walk daily.

Guest: Today we went to the municipality to ask them.

Guest: The track for walking so do not sit, like when you go to the mosque do not get there in the car, walk, walk and never give up, do not depend on the car never; I was walking on my feet throughout the month of Ramadan.

Guest: Every day I install the treadmill in the room even if I do not walk, if a person is shy buy him a treadmill. It is great specifically for the hands and legs. I think walking on the treadmill it is enough.

Guest: No.

Guest: which aspect do you mean?

Guest: No, there is no difference at all.

Guest: Nothing.

Guest: Just an observer of the exercise with me at the gym from 10 am because I wake up at 10 am in the afternoon, I mean from 10 to 12, I do not have a problem after the evening praying, I do not have a problem at all.

Guest: Perfect.

Guest: Anything, because I do not have lunch for more than 6 or 7 years, fruits are enough for me.

Guest: I do not have lunch, I break the fast at 11 or 10 am then do not have lunch because I depend on fruit, arugula, parsley, cabbage, besides, a kiwi and a half of an orange, this is my food, this is me.

I may take a little snack in the afternoon, and sit to 9 or 10, I do not sleep from 9 or 10 to 1 there is no sleep. This is my system and I think it is good.

Guest: Yes.

Guest: That is good.

Guest: Ok, I am free I do not have a job.

Guest: I will encourage that. Excellent.

Guest: They are organized.

Guest: I mean a complete group.

Guest: great, I will be a supporter.

Guest: Yes, I have no problem.

Guest: yes, I do and sport as well, both of them.

Guest: Yes, I want to say at the beginning of diabetes you can adopt perfectly, because after 20 years -with all due respect many people-nerves become weak and intercourse will be weak, it goes to the extreme.

Guest: Let me tell you why? He who ignores diabetes will be over, but he who pays perfect attention to it, I think would live 30 years without problems.

Guest: No. The most important thing is olive oil. It is more important than the foodstuffs called industrial oils. Stay far away from them! No matter how expensive it could be olive oil the best for your health.

Guest: And what about salads? I think salads are the officially most important factor.

Guest: You are welcome.
