# Supplementary material for: Cultural adaptation of self-management of type 2 diabetes in Saudi Arabia (qualitative study)
Source: PLoS One. 2020 Jul 28;15(7):e0232904. doi: 10.1371/journal.pone.0232904 (PMC7386581; doi:10.1371/journal.pone.0232904)
Supplement: S1 Appendix — (DOCX) [file pone.0232904.s014.docx]

# Appendix 1. Moderator guide

**Moderator Questions Guide in English**

The questions guide is related to the broad line of the DESMOND program and aim to discuss with patients and their health professional their thoughts about diabetes and DSME. The art of the moderator is to ask almost the same questions to the health professional and the patients with a minimum alteration.

**PATIENTS’ INTERVIEWS**

| **Questions** |
| --- |
| **Questions learnt from Previous Quantitive Study, e.g.**  How about your fruit and vegetables consumption? Have you always being keen on consuming fruits and vegetables?  Do you eat fast food? How frequently? Did you cut down on fast food after you became aware of your diabetes?  Do you walk or do any physical activity? How frequently? Are these new habits?  What is the main source of learning about your diabetes? |
| Is it easy for you to take care of your diabetes, do you need help?  Hint: Equipment, family help/support, professional support. |
| Who can help you? |
| What do you know about diabetes, and how do you feel about having diabetes? |
| What are the challenges you have because of diabetes?  Hint: concerns, safety, activity, food and social life. |
| How do you get advice about what is healthy food, and what would be healthy to eat for your diabetes?  Hint: Traditional food, fast food, vegetables and fruits, low calories. |
| Do you think you (or your family) need advice on how your diabetes cannot prevent you from joining the family food? |
| What about physical exercises, do you do any? Would you like to receive some advice on how and when and where you would be able to do this?  Hint: walking, swimming, gym, sport, fear of injury |
| What would prevent from doing regular exercises? Do you think you need some guidance on this? What type of guidance should it be?  Hint: Space, time, safety, cultural barrier. |
| Is it easy for you to walk outdoor? Do you like walking? Is there is a way by which you can be encouraged to walk?  Hint: Advice, arranging walking groups. |
| Is it easy to quit smoking? Do you need help to quit? |
| Is there something different or special in terms of the culture or the family? |
| If a program could be developed that was designed especially for you, to help you eat and live healthy, what would it look like? |
| What about mobile phone? Can they help? Would you like to receive updates and advice texts or phone calls?  Hint: information, new skills. |
| What else would you like in a program? |
| What are your recommendations for getting people here involved in self-management program? |
| Consider everything that has been said today. Is there anything that anyone would like to add? |

**FOCUS GROUPS**

| **Questions (highlights and start lines)** |
| --- |
| For example: (patients with type 2 diabetes can be dependent in their doctor and health carers, talk to me about the patient journey, please). The modulator will be looking for answers for the following questions in the conversation:   - How frequently do they see their patients? - Do they have enough time to explain to them how to take care of their diabetes? - How accessible are they to their patients? |
| For example :( there must be an appointment system in the centre, which dictates how and when the patient will see you, and patient will have to manage their own diabetes themselves in between, Am I correct?). The modulator will be looking for answers for the following questions in the conversation:   - How do they follow-up their patient and their progress in controlling their diabetes? - What medium they believe to be most efficient for increasing the awareness of their patients? |
| For Example: (I’m not sure if you know about self-management education programmes such as DESMOND or DSME, is there is a way that a patient can become independent and responsible for managing their condition?). The modulator will be looking for answers for the following questions in the conversation: |
| - How much do they know about DESMOND? Do they think it is effective?) - How and if can DESMOND be more suitable to their patients? - What else would they like to be in an educational programme to their patients? - What are your recommendations for getting people here involved in self-management programme? |
| For example: (consider everything that has been said today. Is there anything that anyone would like to add?). The modulator will be looking for answers for the following questions in the conversation:  Where they engaged enough?  Do they want to see some actual change happening?  Are they keen for a self-management programme to be introduced? |
